# Supplementary material for: Glucose transporter 10 modulates adipogenesis via an ascorbic acid-mediated pathway to protect mice against diet-induced metabolic dysregulation
Source: PLoS Genet. 2020 May 26;16(5):e1008823. doi: 10.1371/journal.pgen.1008823 (PMC7274451; doi:10.1371/journal.pgen.1008823)
Supplement: S1 File — (PDF) [file pgen.1008823.s001.pdf]

SUPPLEMENTARY DATA

Materials and Methods:

Genetic association studies: single-nucleotide polymorphism (SNP) selection, clinical data collection, and genotyping platform

We searched the GWAS Central database (http://www.gwascentral.org) for associations of phenotypes near/within the *Slc2a10* (GLUT10) gene. Search criteria included a *p*-value threshold of 0.05 and 10 kb-flanking regions. SNPs within the region were identified from meta-analysis of a glucose and insulin-related traits study [1], a 2-h glucose challenge study[2], an adult body mass index study in a British population [3] , and a T2D study[4] as listed in S1 Table.

Measurement of food intake and metabolic rate

Mice were housed individually for measurement of food intake using Tecniplast® Metabolic Cage (TECNIPLAST S.p.A, Via I Maggio, Italy), or their metabolic rate was measured using the CLAMS-home cage (CLAMS-HC) system (Columbus Instruments, Columbus, OH, USA) in the Taiwan Mouse Clinic at Academia Sinica. The first readings were taken after a 48-h acclimation period. Heat production, RER, oxygen consumption rate (VO2), and carbon dioxide production (VCO2) rates were determined. VO2, VCO2, and heat were measured every 17 min during a 76-h period at the indicated temperature and were normalized to body weight.

Mice

Glut10^{G128E} mice were backcrossed for more than 10 generations from a C3H background onto the C57BL/6J background, which is widely used in metabolic disease research [5, 6]. *Glut10*^{G128E} mice on a C57BL/6J background had similar phenotypes to those previously observed in *Glut10*^{G128E} mice on the C3H background. *Glut10*^{G128E} mice and their wild-type littermates (WT) control mice were produced from heterozygous breeding pairs. Animals were weaned at 3 weeks of age onto CD and genotyped at 4 weeks of age. *Glut10*^{G128E} and WT mice were randomly assigned to CD or HFD at 5 weeks of age. Male mice were used in this study. No data were excluded in the analysis. Mice were weighed weekly. For fasting blood glucose measurement, GTT, and ITT studies, blood samples were collected from mice at indicated time points using a 30-gauge needle to puncture the tip of the tail. At the conclusion of CD and HFD-feeding, animals were killed by CO2 asphyxiation. Blood was collected from cardiac puncture for blood chemistry and adipokines assays, and tissues were dissected immediately, weighed, and snap frozen in liquid nitrogen (for RT-PCR or western blot assays) or fixed (for histological analysis and immunohistochemistry studies).

Fluorescent microbead perfusion for vasculature studies

The functional architecture of the adipose vasculature in mice was examined by intravenous injection of fluorescent microbeads as described [7]. In brief, mice were injected with 150 µl of fluorescent microbeads in PBS (red fluorescence, 0.1 µm in diameter microbeads, Invitrogen) in their tail vein, anaesthetized 5-10 min later, and then perfused through the heart with 6 ml of 1:10 PBS dilution of fluorescent microbeads. Tissues were then dissected, embedded, sectioned, and analyzed of blood vessel density using fluorescence microscopy

Isolation of stromal vascular fraction (SVF) cells

SVF cells were freshly isolated from eWATs of WT and *GLUT10*^{G128E} male mice (at age 6–8 weeks) as previously described [8].

OCR measurements

OCR was measured using the Seahorse XF24 extracellular flux analyzer as previously described [9].

ROS measurements

Intracellular and mitochondrial ROS were measured using 2′,7′-dichlorodihydrofluorescein

diacetate (DCFH-DA; Sigma-Aldrich, St. Louis, MO, USA) and MitoSox Red (Thermo Fisher, Miami, FL, USA), respectively.

Primer sequences used for PCR:

Gene	Forward primer (5'- 3')	Reverse Primer (5'- 3')
**RT-qPCR**		
36B4	TCCAGGCTTTGGGCATCA	CTTTATCAGCTGCACATCACTCAGA
β-actin	CCAACCGTGAAAAGATGACC	ACCAGAGGCATACAGGGGACA
Gapdh	ATGACCACAGTCCATGCCATC	GAGCTTCCCGTTCAGCTCTG
Emr1	TGTAACCGGATGGCAAACT	CTGTACCCACATGGCTGATG
Tnfα	CACAGAAAGCATGATCCGCGACGT	CGGCAGAGAGGAGGTTGACTTTCT
Tgf β	CTCCCGTGGCTTCTAGTGC	GCCTTAGTTTGGACAGGATCTG
Il-6	TCCAGTTGCCTTCTTGGGAC	GTACTCCAGAAGACCAGAGG
Adiponectin	CCTGGAGAGAAGGGAGAGAAA	GTTCCCAATGTACCCATTCG
Leptin	CCGTGGCTTTGGTCCTATCTG	AGGCAAGCTGGTGAGGATCTG
Pgc1a	CCCTGCCATTGTTAAGACC	TGCTGCTGTTCCTGTTTTC
Pgc1β	TGCGGAGACACAGATGAAGA	GGCTTGTATGGAGGTGTGGT
Pparr	GCCCTTTGGTGACTTTATGG	CAGCTGGCGACATACAGTAC
C/Ebpa	AGCTTACAACAGGCCAGGTTT	CGGCTGGCCGACATACAGTAC
Dlk1/Pref1	GACCCACCCTGTGACCCC	CAGGCAGCTCGTGCACCCC
Fabp4/aP2	CCCTGCGTGATCAATGGT	CACAGAAGTTGCCTGAGAAGC
Slc2a1	TCAACACGGCCTTCACTG	CACGATGCTCAGATAGGACAT
Slc2a2	TCTTCACGGCTGTCTCTGTG	AATCATCCCGGTTAGGAACA
Slc2a3	ATGGCAGTGGCTGGTTGTTC	TCCTGAGCTGAAGAGAATGTC C
Slc2a4	GGTTGTCTTGACCCCTCCAG	TTCGGGTTTAGCACCCTTCC
Slc2a6	TTGGTGCTGTGAGGCT	TGGCACAAACTGGACGTA
Slc2a8	GCTCCTCATGTCAGAGATCTT C	GGCTGTGATTTGTTCCAGAGTC
Slc2a9	TGCTTCCTCGTCTTCGCCACAATA	CTCTTGGCAAATGCCTGGCTG ATT
Slc2a10	CCTGGCTTTCATCTACCTACTTGTT	ATGGTACTGAATACCGATGCGC
Slc2a12	TGAGTTTGTCCTACACGCTCCTT	GGCAATTTCCGCAATGTACA
Slc23a1	CAGCAGGGACTTCCACCA	CCACACAGGTGAAGATGGTA
Slc23a2	CAGCAGGGACTTCCACCA	CATCTGTGCGTGCATAGTAGC
**ChIP-PCR**		
Pparg	CCTGAATTTACCCGAGCTGA	TTATCTCGGAGGCGGTAAGA
Cebpa	ACTGGCGCCTTCGATCCGAGA	AGCTTCGGGTCGCGAATGGC

Antibodies used for western blotting and immunohistochemistry

Antibodies

Secondary antibody	Anti	Cat. #	Company	WB	IHC
Anti-Mouse IgG (Fc Specific)	mouse	A0168	Sigma-Aldrich	1:5,000	
Anti-Rabbit IgG (whole molecule)	rabbit	A0545	Sigma-Aldrich	1:10,000	1:100
**Primary antibody**	**Source**	**Cat**	**Company**		
Beta-actin	rabbit	GTX109639	Genetex	1:10,000	
DLK1	rabbit	10636-1-AP	Proteintech	1:500	
FABP4	rabbit	12802-1-AP	Proteintech	1:7,000	
C/EBPα	rabbit	sc-61	Santa Cruz	1:400	
PPARγ	rabbit	sc-7196	Santa Cruz	1:800	
Collagen I	rabbit	ab34710	Abcam,	1:1,000	

CD68	rabbit	A13286	Abclonal,	1:50		1:50
Collagen I	rabbit	ab34710	Abcam,	1:1,000		

A position map is shown for the Proteome Profiler Mouse Adipokine Array kit from R&D Systems.

	1	2	3	4	5	6	7	8	9	10	11	12	13	14	15	16	17	18	19	20	21	22	23	24
A	PC																						PC	
B	Adiponectin		AgRP		ANGPTL3		CRP		CD26		ESM-1		Fetuin A		FGF-1		FGF-21		HGF		ICAM-1		IGF-I	
C	IGF-II		IGFBP-1		IGFBP-2		IGFBP-3		IGFBP-5		IGFBP-6		IL-6		IL-10		IL-11		Leptin		LIF		Lipocalin-2	
D	MCP-1		CSF-1		OSM		PTX 2		PTX 3		Pref-1		RAGE		RANTES		RBP4		Resistin		PAI-1		TIMP-1	
E	TNF-α		VEGF-A																					
F	PC																						NC	

The adipokine signals with observable changes in Fig. 3I are shaded grey in the table.

References

1. Dupuis J, Langenberg C, Prokopenko I, Saxena R, Soranzo N, Jackson AU, et al. New genetic loci implicated in fasting glucose homeostasis and their impact on type 2 diabetes risk. Nat Genet. 2010;42(2):105-16. Epub 2010/01/19. doi: 10.1038/ng.520. PubMed PMID: 20081858; PubMed Central PMCID: PMCPMC3018764.
2. Saxena R, Hivert MF, Langenberg C, Tanaka T, Pankow JS, Vollenweider P, et al. Genetic variation in GIPR influences the glucose and insulin responses to an oral glucose challenge. Nat Genet. 2010;42(2):142-8. Epub 2010/01/19. doi: 10.1038/ng.521. PubMed PMID: 20081857; PubMed Central PMCID: PMCPMC2922003.
3. Strachan DP, Rudnicka AR, Power C, Shepherd P, Fuller E, Davis A, et al. Lifecourse influences on health among British adults: effects of region of residence in childhood and adulthood. Int J Epidemiol. 2007;36(3):522-31. Epub 2007/01/27. doi: 10.1093/ije/dyl309. PubMed PMID: 17255346.
4. Scott LJ, Mohlke KL, Bonnycastle LL, Willer CJ, Li Y, Duren WL, et al. A genome-wide association study of type 2 diabetes in Finns detects multiple susceptibility variants. Science. 2007;316(5829):1341-5. Epub 2007/04/28. doi: 10.1126/science.1142382. PubMed PMID: 17463248; PubMed Central PMCID: PMCPMC3214617.
5. Cheng CH, Kikuchi T, Chen YH, Sabbagha NG, Lee YC, Pan HJ, et al. Mutations in the SLC2A10 gene cause arterial abnormalities in mice. Cardiovasc Res. 2009;81(2):381-8. PubMed PMID: 19028722.
6. Lee YJ, Ko EH, Kim JE, Kim E, Lee H, Choi H, et al. Nuclear receptor PPARgamma-regulated monoacylglycerol O-acyltransferase 1 (MGAT1) expression is responsible for the lipid accumulation in diet-induced hepatic steatosis. Proc Natl Acad Sci U S A. 2012;109(34):13656-61. Epub 2012/08/08. doi: 10.1073/pnas.1203218109. PubMed PMID: 22869740; PubMed Central PMCID: PMCPMC3427113.
7. Jonker JW, Suh JM, Atkins AR, Ahmadian M, Li P, Whyte J, et al. A PPARgamma-FGF1 axis is required for adaptive adipose remodelling and metabolic homeostasis. Nature. 2012;485(7398):391-4. doi: 10.1038/nature10998. PubMed PMID: 22522926; PubMed Central PMCID: PMC3358516.
8. Soukas A, Socci ND, Saatkamp BD, Novelli S, Friedman JM. Distinct transcriptional profiles of adipogenesis in vivo and in vitro. J Biol Chem. 2001;276(36):34167-74. Epub 2001/07/11. doi: 10.1074/jbc.M104421200. PubMed PMID: 11445576.

9. Syu YW, Lai HW, Jiang CL, Tsai HY, Lin CC, Lee YC. GLUT10 maintains the integrity of major arteries through regulation of redox homeostasis and mitochondrial function. Hum Mol Genet. 2018;27(2):307-21. doi: 10.1093/hmg/ddx401. PubMed PMID: 29149261.
