# Supplementary material for: Glucose transporter 10 modulates adipogenesis via an ascorbic acid-mediated pathway to protect mice against diet-induced metabolic dysregulation
Source: PLoS Genet. 2020 May 26;16(5):e1008823. doi: 10.1371/journal.pgen.1008823 (PMC7274451; doi:10.1371/journal.pgen.1008823)
Supplement: S1 Table — (PDF) [file pgen.1008823.s002.pdf]

S1 Table. SNPs in *SLC2A10* region associated with BMI, glucose tolerance, insulin resistance or T2D-related phenotypes in independent human populations and studies

SNP	Phenotype	*P*-value	Study and dataset
rs1073137	Fasting insulin-related: homeostatic model assessment of insulin resistance	0.0035	GWAS of glycemic traits (HGVST463)
rs3091433	Fasting insulin-related: homeostatic model assessment of insulin resistance	0.0030	GWAS of glycemic traits (HGVST463)
rs4809599	Fasting glucose-related: homeostatic model assessment of beta-cell function	0.0397	GWAS of glycemic traits (HGVST463)
rs11086177	Fasting glucose-related: homeostatic model assessment of beta-cell function	0.0293	GWAS of glycemic traits (HGVST463)
rs4239646	Fasting glucose-related: homeostatic model assessment of beta-cell function	0.0412	GWAS of glycemic traits (HGVST463)
rs4810546	Fasting glucose-related: homeostatic model assessment of beta-cell function	0.0407	GWAS of glycemic traits (HGVST463)
rs6018026	Fasting glucose-related: homeostatic model assessment of beta-cell function	0.0432	GWAS of glycemic traits (HGVST463)
rs6124859	Fasting glucose-related: homeostatic model assessment of beta-cell function	0.0490	GWAS of glycemic traits (HGVST463)
rs13038390	Fasting glucose-related: fasting plasma glucose	0.0157	GWAS of glycemic traits (HGVST463)
rs8119705	Fasting glucose-related: fasting plasma glucose	0.0138	GWAS of glycemic traits (HGVST463)
rs11550540	Fasting glucose-related: fasting plasma glucose	0.0092	GWAS of glycemic traits (HGVST463)
rs13044870	Fasting glucose-related: fasting plasma glucose	0.0098	GWAS of glycemic traits (HGVST463)
rs2143044	Fasting glucose-related: fasting plasma glucose	0.0474	GWAS of glycemic traits (HGVST463)
rs3091619	Fasting glucose-related: fasting plasma glucose	0.0150	GWAS of glycemic traits (HGVST463)
rs4810537	Fasting glucose-related: fasting plasma glucose	0.0088	GWAS of glycemic traits (HGVST463)
rs971759	Fasting glucose-related: fasting plasma glucose	0.0103	GWAS of glycemic traits (HGVST463)
rs12479748	Fasting glucose-related: fasting plasma glucose	0.0501	GWAS of glycemic traits (HGVST463)
rs2425903	Fasting glucose-related: fasting plasma glucose	0.0445	GWAS of glycemic traits (HGVST463)
rs6017999	Fasting glucose-related: fasting plasma glucose	0.0416	GWAS of glycemic traits (HGVST463)
rs6018020	Fasting glucose-related: fasting plasma glucose	0.0380	GWAS of glycemic traits (HGVST463)
rs6090546	Fasting glucose-related: fasting plasma glucose	0.0488	GWAS of glycemic traits (HGVST463)
rs2694899	Type II diabetes	0.0020	GWAS of type II diabetes mellitus (HGVST5)
rs6018037	Two-hour glucose challenge	0.0494	GWAS of glucose levels 2 hours after an oral glucose challenge (HGVST433)
rs6094461	Two-hour glucose challenge	0.0384	GWAS of glucose levels 2 hours after an oral glucose challenge (HGVST433)
rs4810537	Body mass index	0.0246	GWAS of adult body mass index in a British population (HGVST308)
rs998422	Body mass index	0.0106	GWAS of adult body mass index in a British population (HGVST308)
rs3091619	Body mass index	0.0432	GWAS of adult body mass index in a British population (HGVST308)
rs998422	Body mass index	0.0170	GWAS of adult body mass index in a British population (HGVST308)
rs3092706	Body mass index	0.0136	GWAS of adult body mass index in a British population (HGVST308)
rs2425902	Body mass index	0.0490	GWAS of adult body mass index in a British population (HGVST308)
rs2425908	Body mass index	0.0155	GWAS of adult body mass index in a British population (HGVST308)

Data were analyzed using GWAS Central database.
