# Supplementary material for: Glucose transporter 10 modulates adipogenesis via an ascorbic acid-mediated pathway to protect mice against diet-induced metabolic dysregulation
Source: PLoS Genet. 2020 May 26;16(5):e1008823. doi: 10.1371/journal.pgen.1008823 (PMC7274451; doi:10.1371/journal.pgen.1008823)
Supplement: S3 Table — (PDF) [file pgen.1008823.s004.pdf]

S3 Table. Variants in the *SLC2A10* region

Variant type	Number of variants
Non coding transcript variant	2404
5’ UTR variant	74
3’ UTR variant	663
Missense variant	486
Noncoding transcript exon variant	298
Intron variant	7829
Coding sequence variant	228
Stop gained	15
Synonymous variant	230
Frameshift variant	25
In-frame insertion	1
In-frame deletion	4
Stop retained variant	3
Start lost	4
Splice region variant	45
Splice donor variant	10
Splice acceptor variant	6

Chromosome 20:46,709,649-46,736,347 (GRCh38:CM000682.2).
Variant classification using Ensemble.
