# Supplementary material for: Glucose transporter 10 modulates adipogenesis via an ascorbic acid-mediated pathway to protect mice against diet-induced metabolic dysregulation
Source: PLoS Genet. 2020 May 26;16(5):e1008823. doi: 10.1371/journal.pgen.1008823 (PMC7274451; doi:10.1371/journal.pgen.1008823)
Supplement: S1 Fig — (A) Schematic model of the structure of GLUT10. (B) Amino acid sequence alignment of GLUT10 in Homo sapiens, Pan troglodytes, Macaca mulatta, Canis lupus familiaris, Bos taurus, Mus musculus and Rattus norvegicus. The yellow highlight indicates the G128 residue is highly conserved in mammals. The conserved domain is indicated in red. The transmembrane domains (TM) 3, 4 and 5 are indicated. (C) The variant effect predicted by Ensembl Variant Effect Predicator. The variant is predicted to be benign. (PDF) [file pgen.1008823.s005.pdf]

A

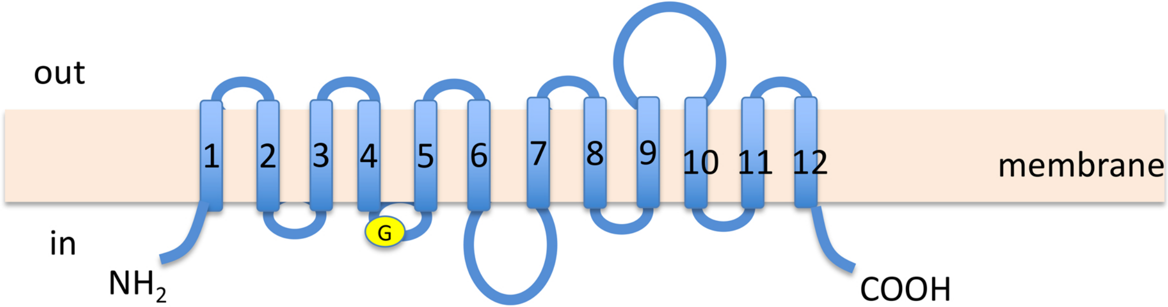

B

```
                                   TM 3                                  TM 4                             TM 5
Homo sapiens.           77 AILGSNLVLLAGSLTLGLAGSLAWLVLGRAVVGFAISLSSMACCIYVSELVGPRQRGVLVSLYEAGITVGILLSYALNYA 156
Pan troglodytes.        77 AILGSNLVLLAGSLTLGLAGSLAWLVLGRAVVGFAISLSSMACCIYVSELXGPRQRGVLVSLYEAGITVGILLSYALNYA 156
Macaca mulatta.         77 AILGSNLVLLAGSLTLGLAGSLTWLVLGRSVVGFAISLSSMACCIYVSELVGPRQRGVLVSLYEAGITVGILLSYALNYA 156
Canis lupus familiaris  77 AILGSNLVLLAGSLSLGLAGSLAWLVLGRLVAGFAISLSSMACCIYVSELVGPRQRGVLVSLYEAGITLGILLSYALNYA 156
Bos Taurus              77 AILGSNLVLLAGSLSLGLAGSLAWLLLGRSVAGFAISLSSMACCIHVSELAGPRQRGVLVALYEAGITVGVLLSYALNYA 156
Mus musculus.           77 AILGSNAVLLAGSLILGLASSLPWLLLGRLSVGFAISLSSMACCIYVSELVGPRQRGVLVSLYEVGITVGILFSYGLNYV 156
Rattus norvegicus.      77 AILGSNVVLLAGSLILGLAGSLPWLLLGRSSVGFAISLSSMACCIYVSELVGPRQRGVLVSLYEVGITVGILFSYGLNYV 156
```

C

Variant ID	Alleles	Global MAF	AA	AA coord	SIFT	PolyPhen	CADD	REVEL	MetaLR	Mutation Assessor
rs1226198239	G/A	-	G/E	128	0.07	0.42	22	0.191	0.304	0.36
