# Supplementary figures and images for: Glucose transporter 10 modulates adipogenesis via an ascorbic acid-mediated pathway to protect mice against diet-induced metabolic dysregulation
Source: PLoS Genet. 2020 May 26;16(5):e1008823. doi: 10.1371/journal.pgen.1008823 (PMC7274451; doi:10.1371/journal.pgen.1008823)

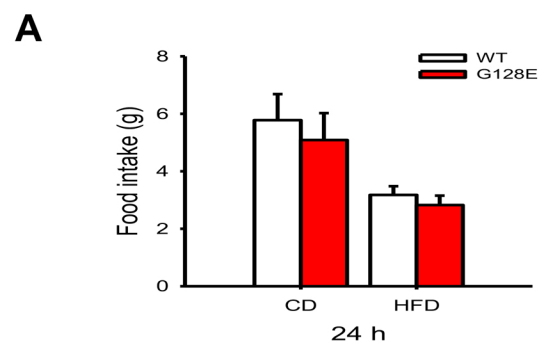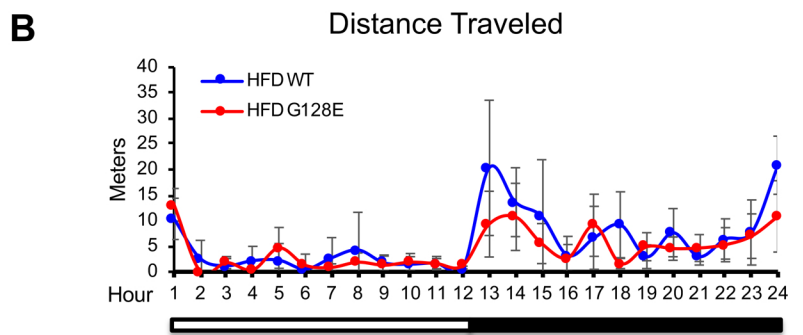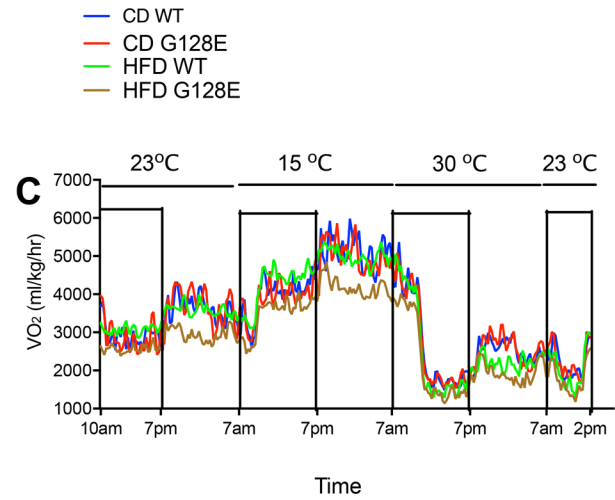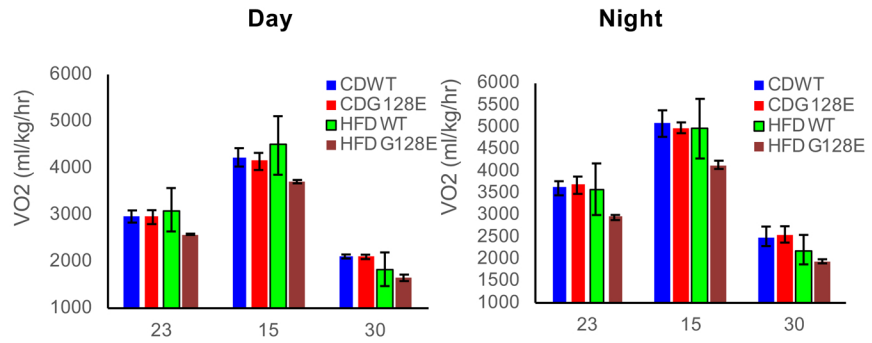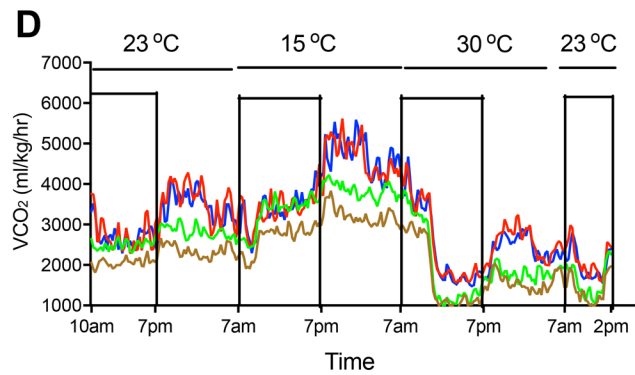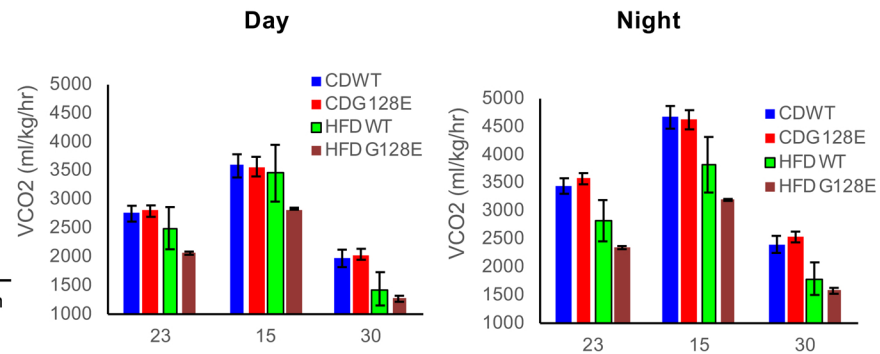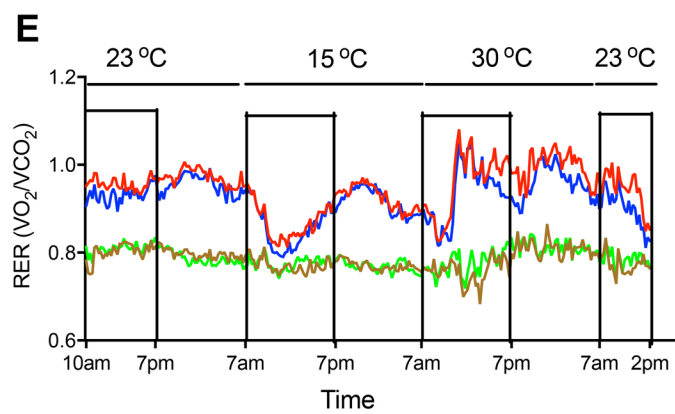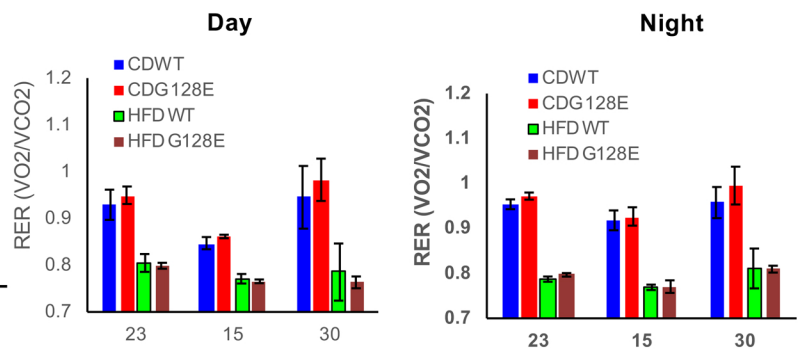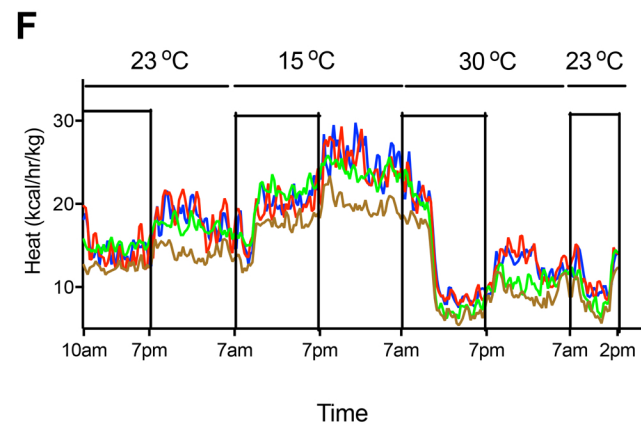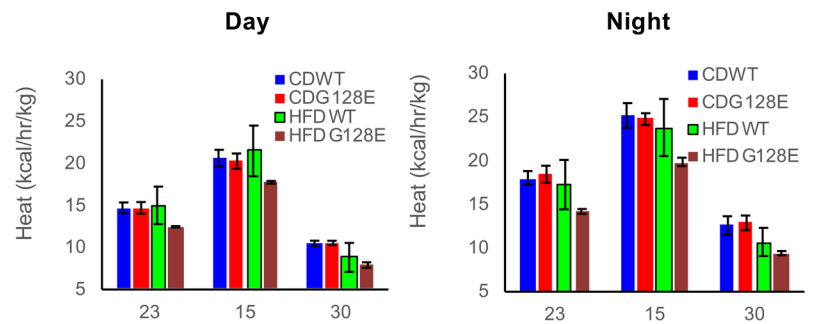

Supplement: S2 Fig — WT and Glut10G128E mice were fed a CD or HFD from 5 to 20 weeks of age. Data were collected from mice at the conclusion of feeding. (A) Average food intake over a 24-h period. (B) Physical activity over a 24-h period; the bar below the graph indicates the light and dark portion of the day. Physical activity was measured in WT and Glut10G128E mice under a HFD using Clever Sys HomeCageScan TM3.0. (C–F) The metabolic indicators at different temperatures over a 79-h period. (C) Consumption of O2, (D) CO2 production, (E) Respiratory Exchange Ratio (RER- an assessment of the metabolic exchange of oxygen for carbon dioxide) = VCO2/VO2, and (F) heat generation were measured by a comprehensive laboratory animal monitoring system (CLAMS). Bar graphs on the left indicate the average values during the light and dark cycle at different temperatures. Error bars, SEM. n = 8 mice in each group. (PDF) [file pgen.1008823.s006.pdf]

**A****eWAT**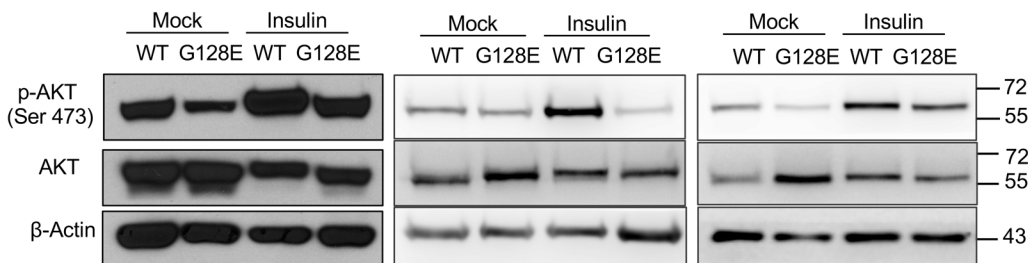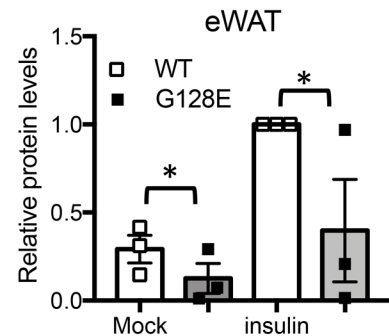**B****Liver**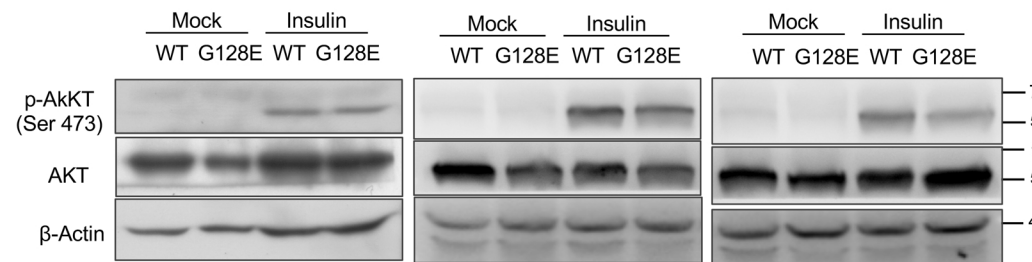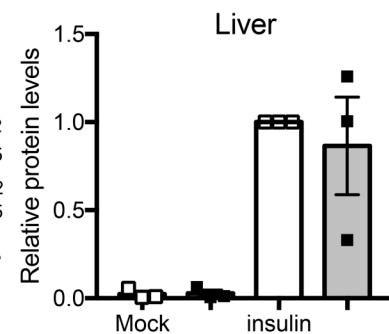**C****Skeletal Muscle**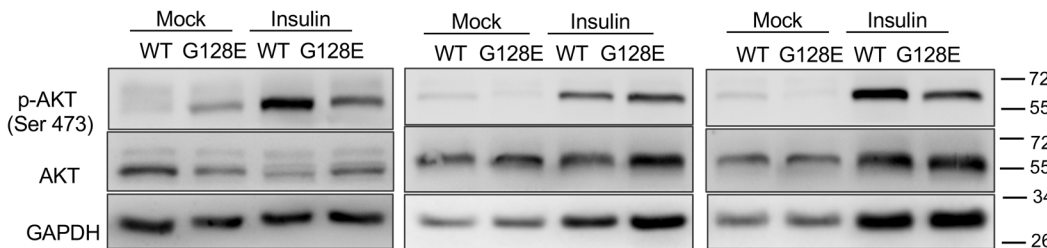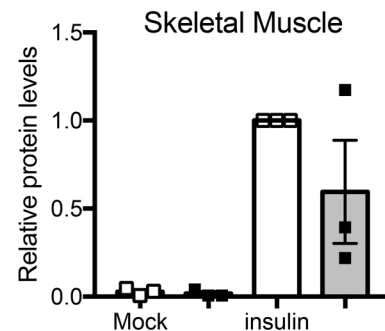

Supplement: S3 Fig — Glut10 and WT mice fed a HFD from 5 to 20 weeks of age were fasted overnight and injected with saline (Mock) or insulin (0.5 U/kg). Mice were killed 30 min after injection. Insulin-induced AKT phosphorylation in (A) eWAT, (B) liver, and (C) skeletal muscle was analyzed by western blotting; relative intensity was quantified (right panels). Error bars, SEM. n = 3 mice in each group. (PDF) [file pgen.1008823.s007.pdf]

**A**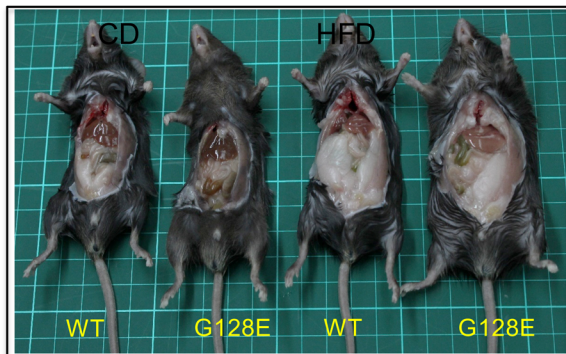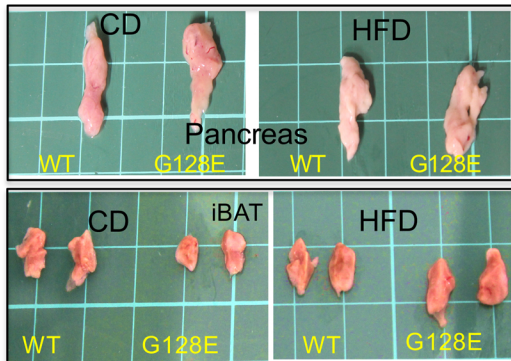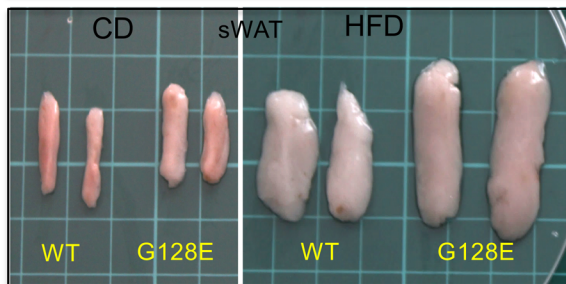**B**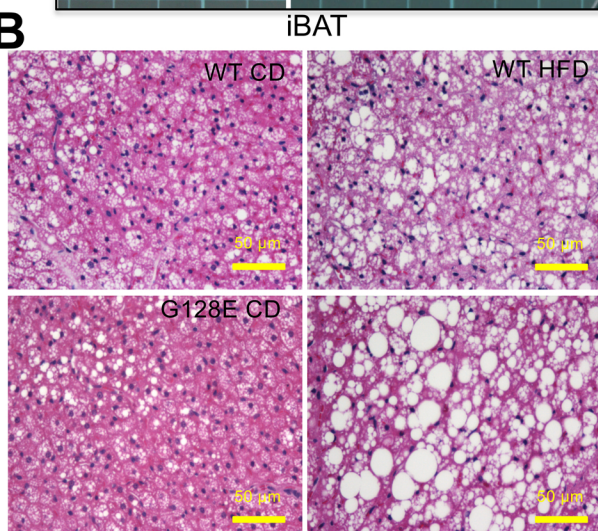**C**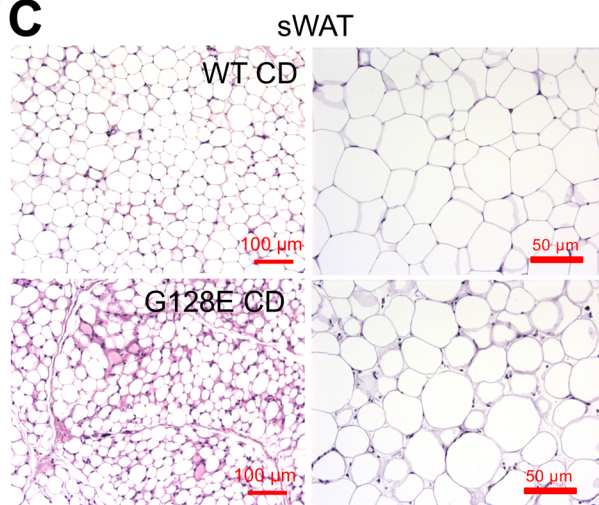**D**

Pancreas islet

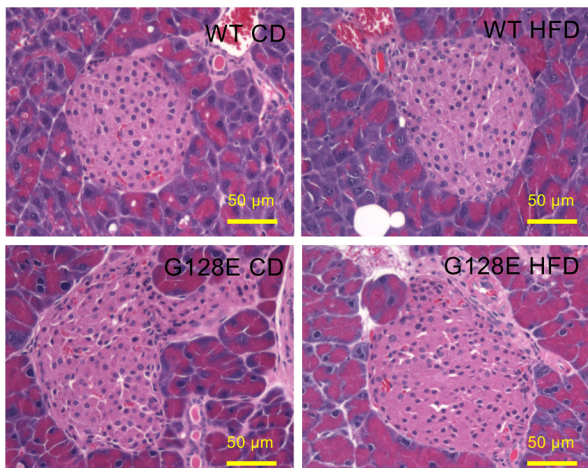

Supplement: S4 Fig — Glut10G128E mice and WT mice were fed a CD or HFD from 5 to 20 weeks of age, after which the mice were killed and tissues collected for analysis. (A) Representative photographs of mice and their tissues. Each square on the green mat is 1 cm × 1cm. (B-D) H&E staining of iBAT, sWAT, and pancreas sections. (PDF) [file pgen.1008823.s008.pdf]

**A**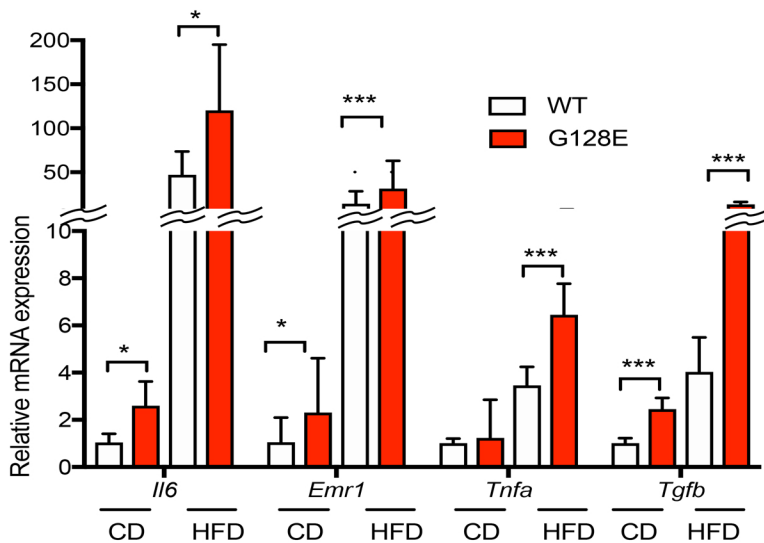**B**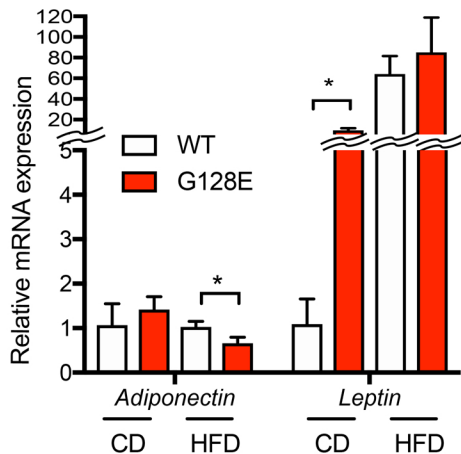**C**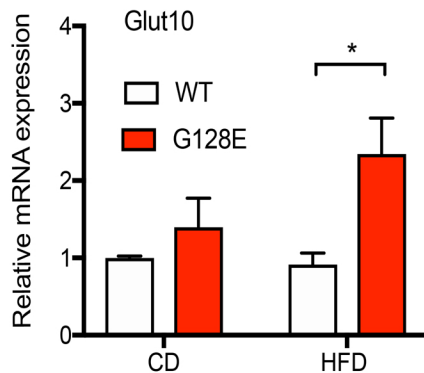

Supplement: S5 Fig — Glut10G128E mice and WT mice were fed with a normal diet (CD) or HFD from 5 to 20 weeks of age. Data were analyzed from the mice at the conclusion of feeding. mRNA expression levels were analyzed in eWAT by RT-PCR. n = 6 mice per group. (A) Genes involved in inflammation and fibrosis. (B) Adiponectin and leptin expression. (C) Glut10 expression. Data are shown as the mean ± SEM. *P < 0.05, **P < 0.01, ***P < 0.001. (PDF) [file pgen.1008823.s009.pdf]

# A Aorta

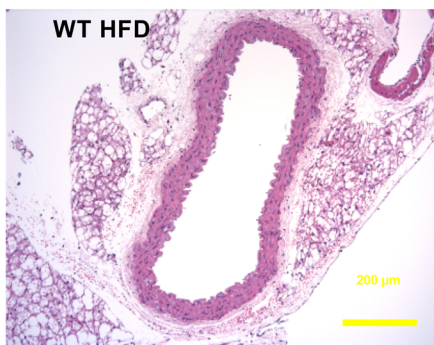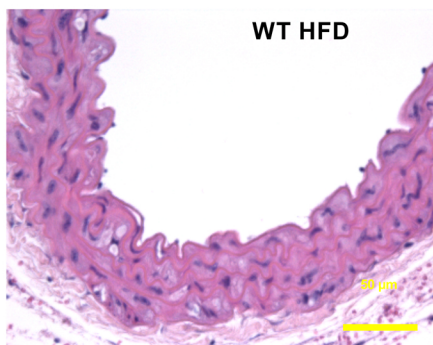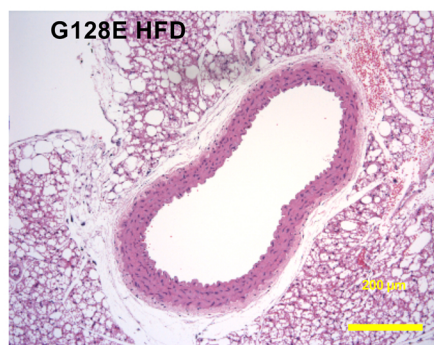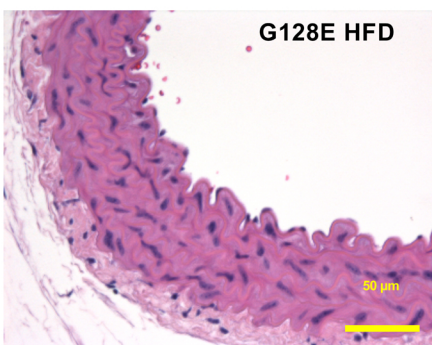

# B eWAT

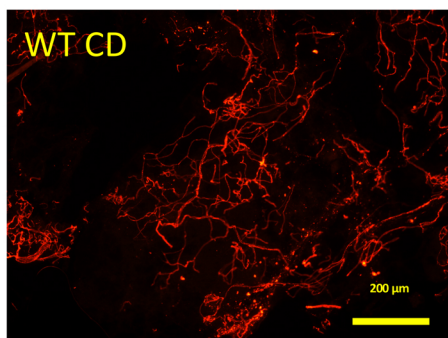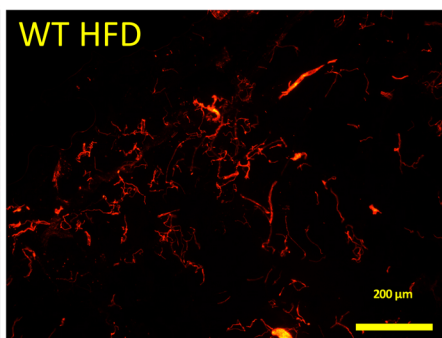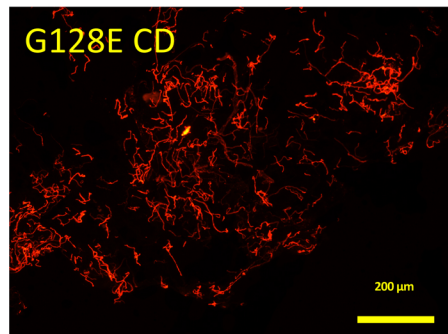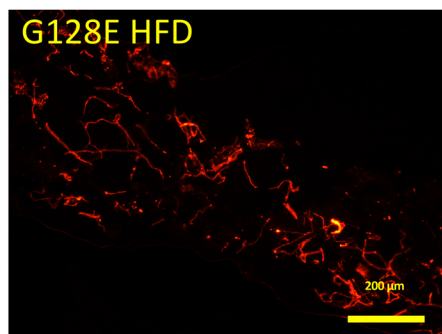

Supplement: S6 Fig — Glut10G128E mice and WT mice were fed a CD or HFD from 5 to 20 weeks of age. Data were collected from the mice at the conclusion of feeding. (A) H&E staining of aorta sections. (B) Fluorescence staining of vascular tissues in eWAT sections. Fluorescence microscopy of eWAT after perfusion with fluorescent microbeads revealed similar vascularization in WT and Glut10G128E mice under both CD and HFD conditions. (PDF) [file pgen.1008823.s010.pdf]

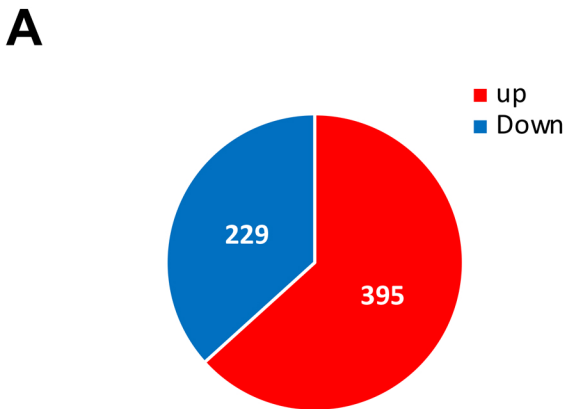

**B** Diseases and Bio Functions

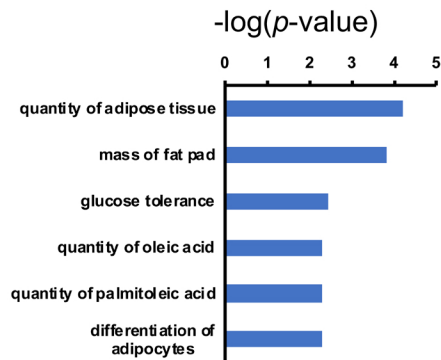

**C** Upstream Regulator

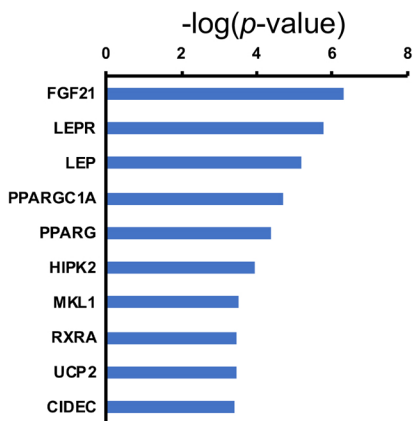

Supplement: S7 Fig — The gene expression profiles in eWATs from WT and Glut10G128E mice at 3 weeks were analyzed by RNA-seq with cutoffs of fold change (FC) ≥ 1.5 and ≤ 0.5. Samples from 8 mice per group were pooled. (A) Pie chart shows 395 genes were upregulated and 229 genes were downregulated in eWAT of Glut10G128E mice. (B and C) The dysregulated genes were analyzed by ingenuity pathway analysis (IPA) for annotations of disease and biological function and upstream regulators. The categories of diseases and bio function showed many dysregulated genes are involved in adipocyte differentiation, development of adipose tissue, lipid metabolism and glucose metabolism. The upstream regulator analysis identified FGF21 (fibroblast growth factor 21, a metabolic pathway regulator), LEPR (leptin receptor, involved in the regulation of body weight), LEP (leptin, involved in the regulation of body weight), PPARGC1A (peroxisome proliferator- activated receptor gamma coactivator 1-alpha, PGC-1α, involved in adipogenesis and energy metabolism), and PPARγ (peroxisome proliferator-activated receptor gamma, a key adipogenic transcription factor) as the top 5 upstream regulators for the dataset. These analyses suggest that GLUT10 regulates adipogenesis, WAT development, adipokine expression and energy metabolism. (PDF) [file pgen.1008823.s011.pdf]

Human

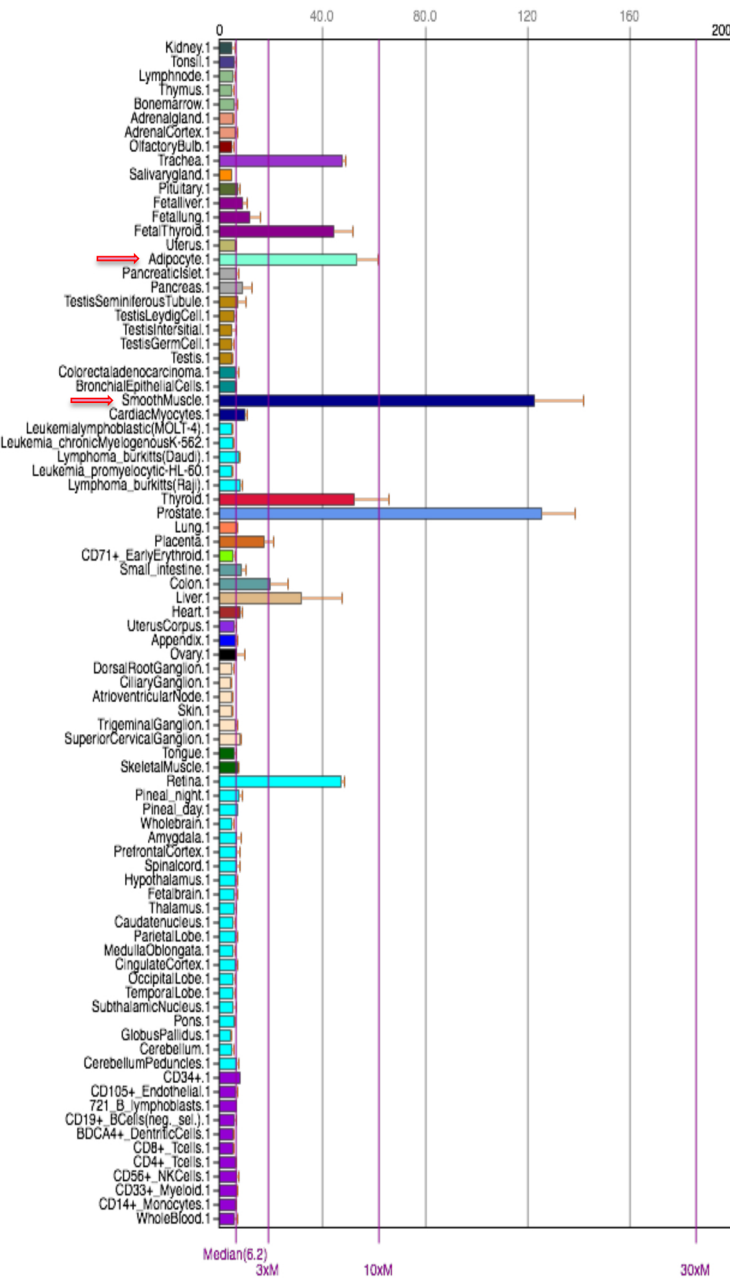

Mouse

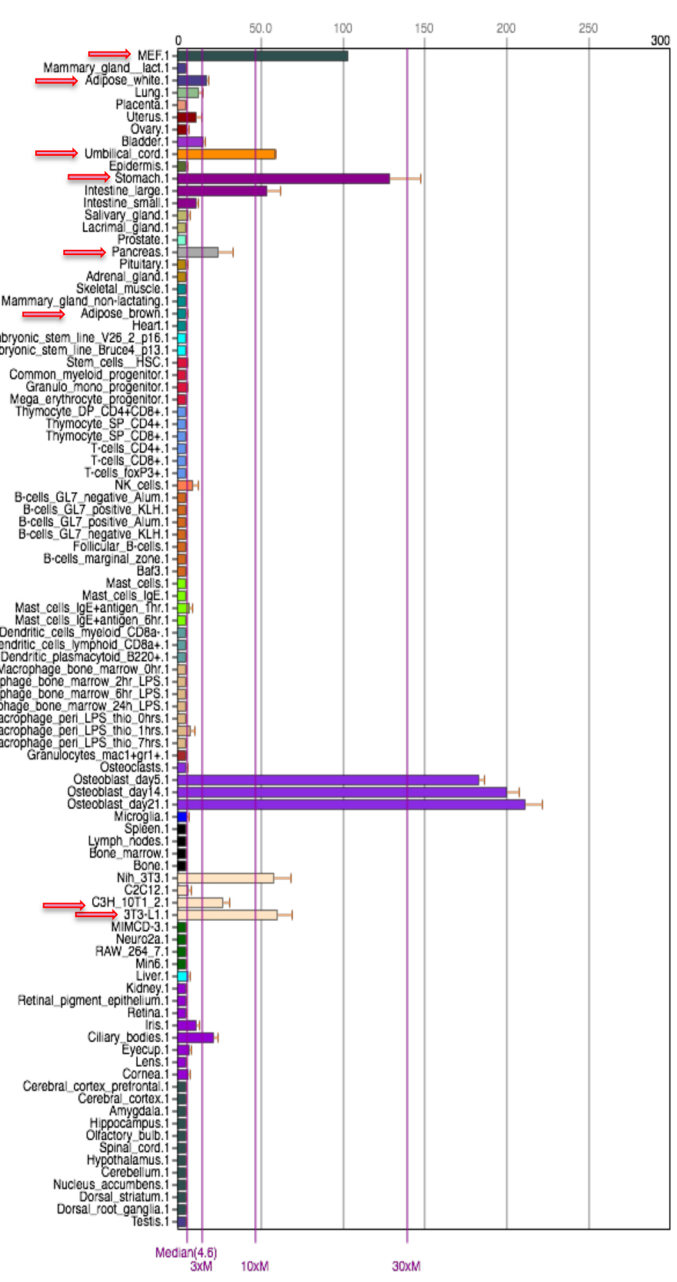

Supplement: S8 Fig — The GDS596 (human) and GDS592 (mouse) expression data sets, available on the NCBI GEO database from Su et al. (https://www.ncbi.nlm.nih.gov/geo/query/acc.cgi?acc=GSE1133), were reanalyzed by BioGPS (http://biogps.org). Relative signal intensity values are depicted in arbitrary units with ranges. The red arrows indicate tissues examined in the current study. (PDF) [file pgen.1008823.s012.pdf]

**A**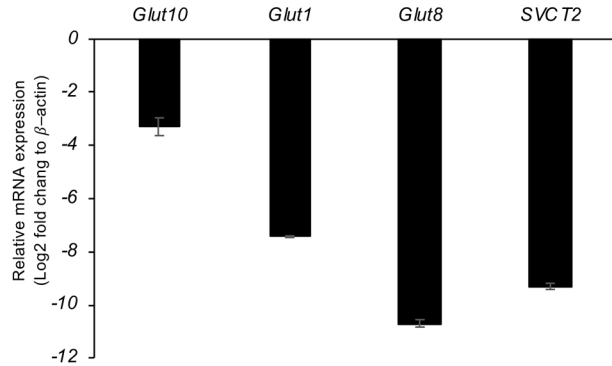**B**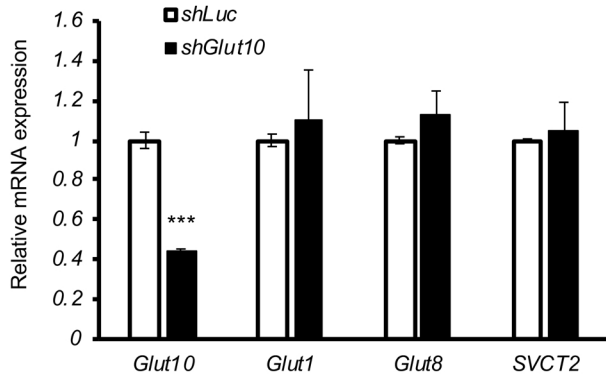

Supplement: S9 Fig — The mRNA expression levels in shLuc and shGlut10 3T3-L1 cells were analyzed by RT-PCR. (A) Only the expression of GLUT10, GLUT1, GLUT8 and SVCT 2 can be detected in 3T3-L1 cells. The relative expression of the expressed transporters was shown in dCT values normalized to β-actin expression. (B) The changes of gene expression in shGlut10 3T3-L1 cells were compared to shLuc 3T3-L1 cells. n = 3 independent experiments. Data are shown as the mean ± SEM. *P < 0.05, **P < 0.01, ***P < 0.001. (PDF) [file pgen.1008823.s013.pdf]

**A**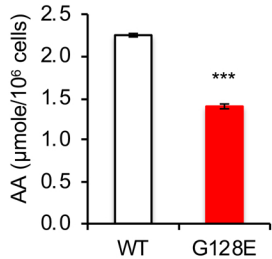**B**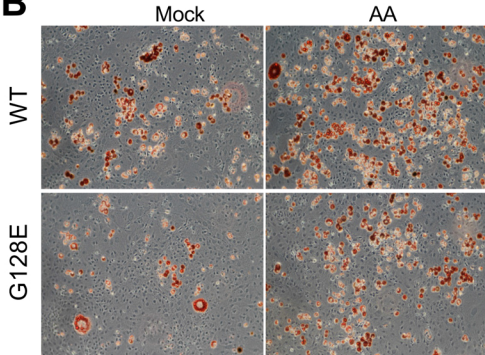**C**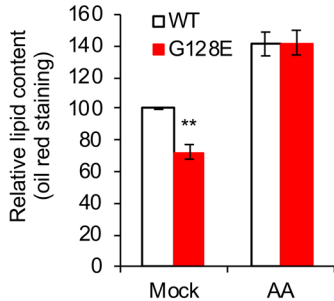

Supplement: S10 Fig — (A) The intracellular ascorbic acid (AA) levels. The MEFs were cultured in medium with 75 μM AA supplemented. (B and C) The MEFs were treated with 75 μM AA or vehicle control (Mock) for 2 days and induced for adipogenic differentiation for 7 days. (B) Representative images of oil red O staining are shown. (C) Quantification of the oil red staining. The data represent mean ± SEM, n = 3 independent experiments per group. *p < 0.05, **p < 0.01, ***p < 0.001. (PDF) [file pgen.1008823.s014.pdf]

**A**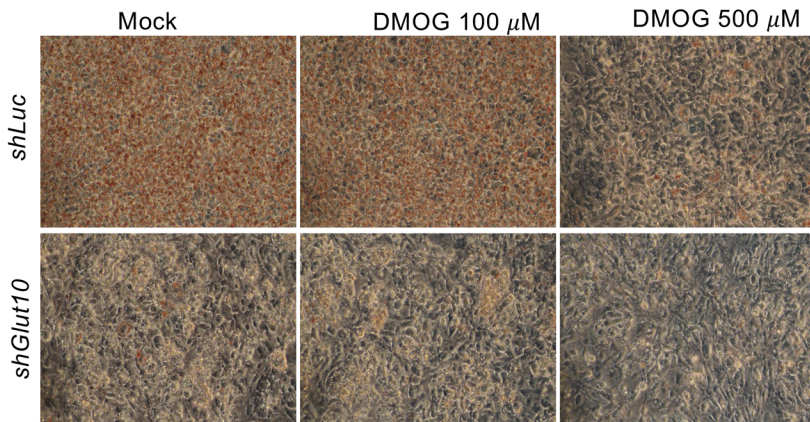**B**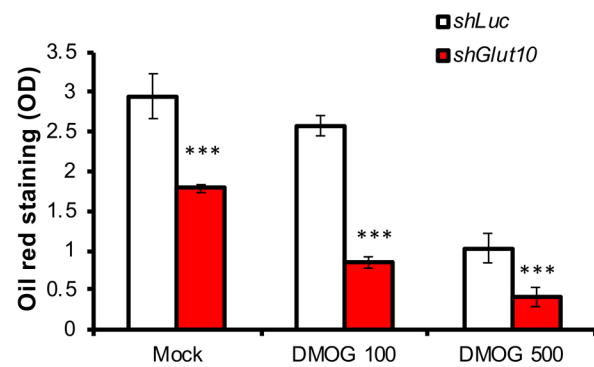**C**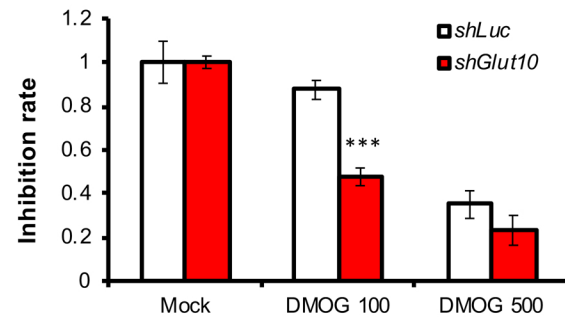**D**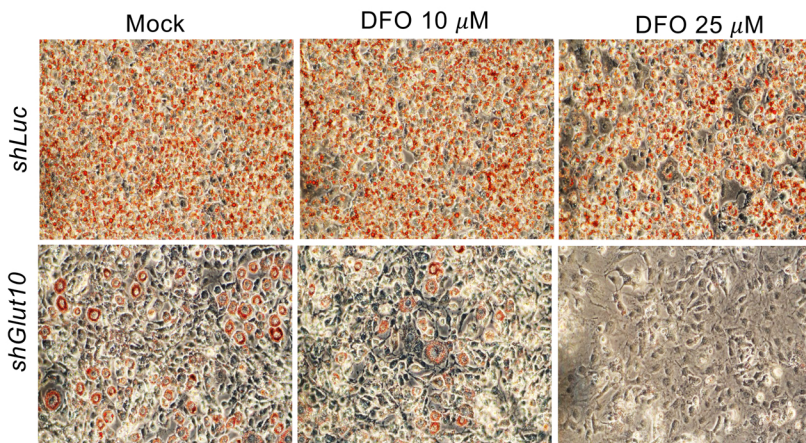**E**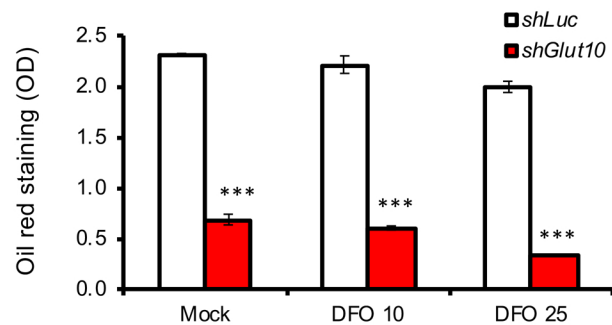**F**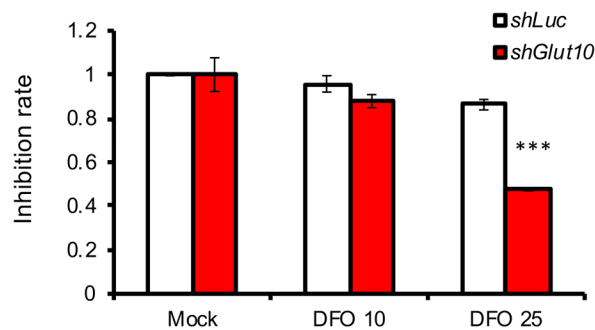

Supplement: S11 Fig — shGlut10 and shLuc 3T3-L1 cells were pretreated with the indicated dose of drug along with vitamin C for 2 days, and adipogenic differentiation was induced for 6 days. (A-C) Cells were pretreated with DMOG or (D-F) DFO. (A, D) Representative images of oil red O staining are shown. (B, C, E, F) Quantification of oil red O staining is shown as the (B, E) OD value and (C, F) relative inhibition rate. Data are shown as the mean ± SEM. n = 3 independent experiments per group. *P < 0.05, **P < 0.01, ***P < 0.001. (PDF) [file pgen.1008823.s015.pdf]

# eWAT

Relative mRNA expression levels

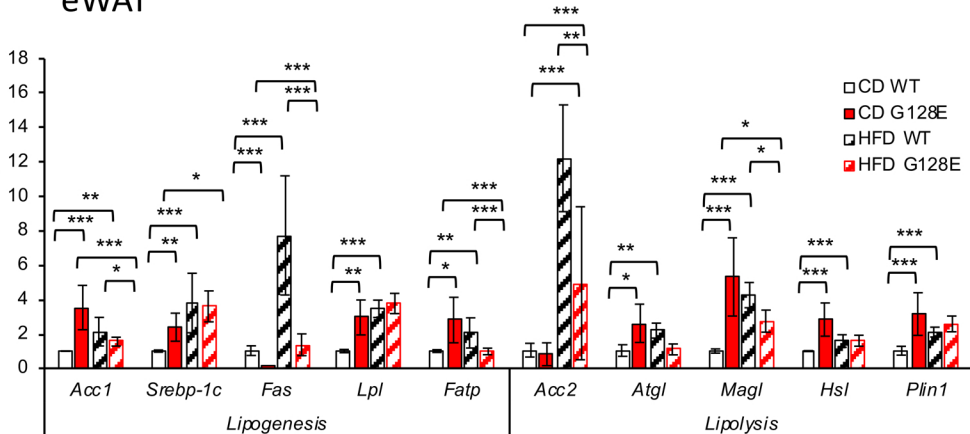

Supplement: S12 Fig — Glut10G128E mice and WT mice were fed with a normal diet (CD) or HFD from 5 to 20 weeks of age. Data were analyzed from the mice at the conclusion of feeding. (A and B) mRNA expression levels were analyzed in eWAT by RT-PCR. n = 6 mice per group. (A) Genes involved in lipogenesis and lipolysis. Data are shown as the mean ± SEM. *P < 0.05, **P < 0.01, ***P < 0.001. (PDF) [file pgen.1008823.s016.pdf]

**A**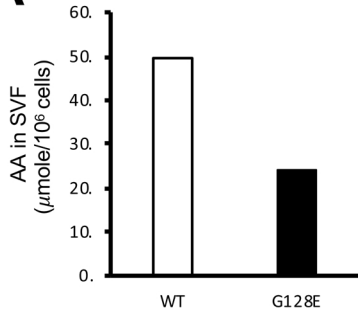**B**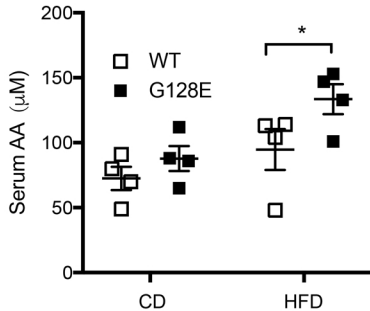

Supplement: S13 Fig — (A) Intracellular ascorbic acid (AA) levels were determined in SVF cells from eWATs of WT and Glut10G128E mice. The eWATs from 7 male Glut10G128E mice and 6 male WT mice (at age 6–8 weeks) were freshly isolated and pooled for AA measurement. (B) Serum ascorbic acid levels were measured from Glut10G128E mice and WT mice fed a CD or HFD from 5 to 20 weeks of age. (PDF) [file pgen.1008823.s017.pdf]

**A**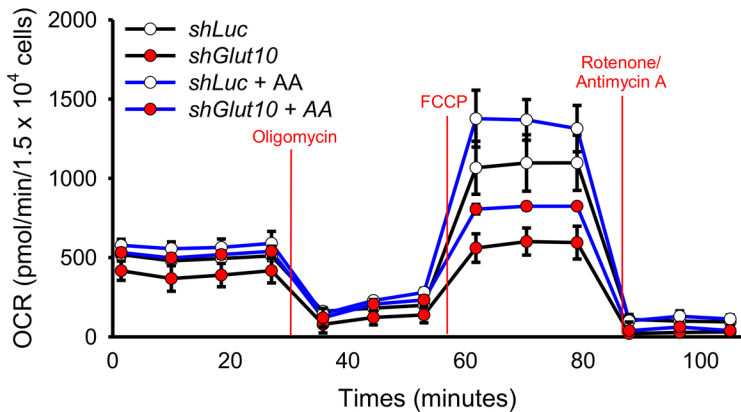**B**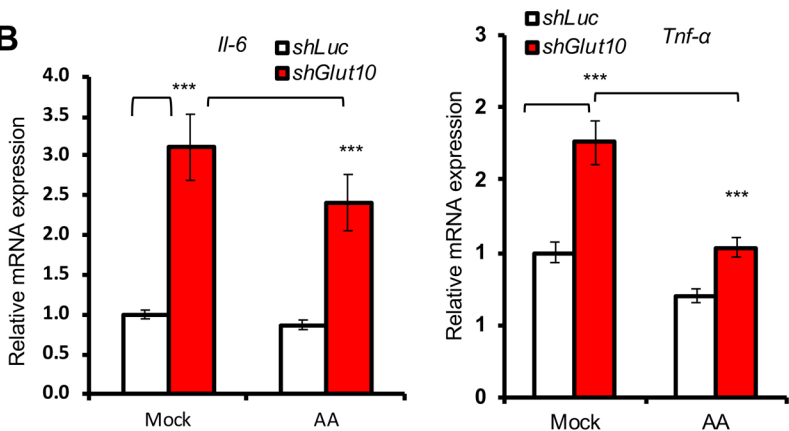

Supplement: S14 Fig — The shGlut10 and shLuc 3T3-L1 preadipocytes were pretreated with 75 μM vitamin C (AA) or vehicle control (Mock) for 2 days. (A) OCR was measured by a Seahorse Bioanalyzer. (B) The mRNA expression of Il-6 and Tnf-α were determined by RT-PCR. Data are shown as the mean ± SEM. *P < 0.05, **P < 0.01, ***P < 0.001. n = 5 per group in A; n = 3 per group in B. *P < 0.05. **P < 0.01, ***P < 0.001. (PDF) [file pgen.1008823.s018.pdf]

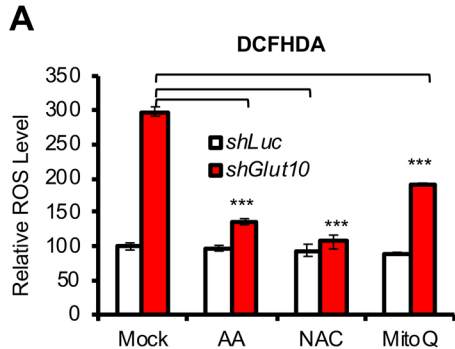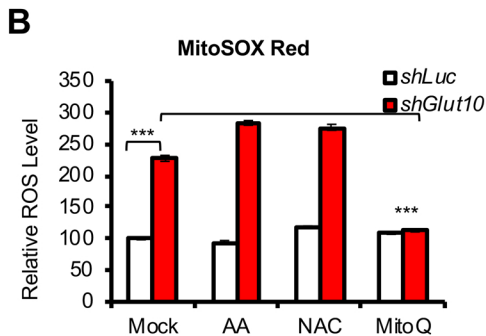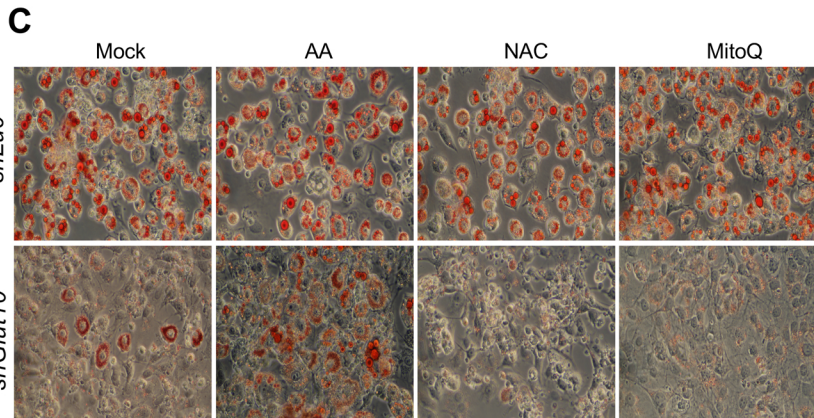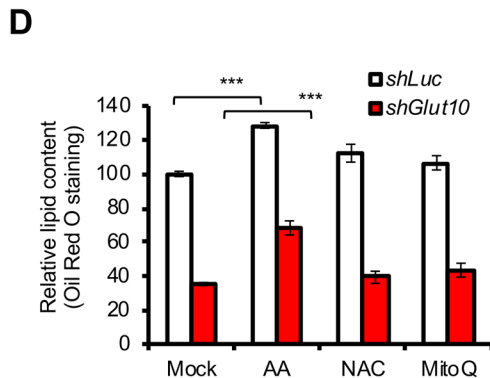

Supplement: S15 Fig — 3T3-L1 cells were pretreated with 75 μM ascorbic acid (AA), 2 mM N-Acetyl-Cysteine (NAC), 50 nM mitochondria-targeted coenzyme Q10 (MitoQ) or vehicle control (Mock) for 2 days and induced for adipogenic differentiation. (A) Intracellular ROS levels and (B) mitochondrial ROS levels were determined in 3T3-L1 preadipocytes after 2 days of treatment. (C) The representative images show oil-red O staining and (D) Oil-red O staining was quantified in 3T3-L1 cells treated for 2 days and after induction of adipogenic differentiation for 6 days. n = 3 per group. (PDF) [file pgen.1008823.s019.pdf]
