# Supplementary material for: Glucose transporter 10 modulates adipogenesis via an ascorbic acid-mediated pathway to protect mice against diet-induced metabolic dysregulation
Source: PLoS Genet. 2020 May 26;16(5):e1008823. doi: 10.1371/journal.pgen.1008823 (PMC7274451; doi:10.1371/journal.pgen.1008823)
Supplement: Attachment — Submitted filename: PLOS point to point cover letter.pdf [file pgen.1008823.s021.pdf]

April 10, 2020

PLOS Genetics (PGENETICS-D-20-00046R1)
Title: Glucose transporter 10 modulates adipogenesis via an ascorbic acid-mediated pathway to protect mice against diet-induced metabolic dysregulation

Gregory S. Barsh
Gregory Copenhaver
Editor-in-Chief
PLOS Genetics

Dear Drs. Barsh and Copenhaver,

Thank you for the opportunity to revise our manuscript "*Glucose transporter 10 modulates adipogenesis via an ascorbic acid-mediated pathway to protect mice against diet-induced metabolic dysregulation*". We appreciate the careful review and constructive suggestions. In this response, we performed additional experiments and changed the text to address editors' and reviewers' questions and concerns.

We are happy to submit the revised manuscript for your consideration. Please find the point-by-point response to the referee's comments in the attached document.

With kind regards,
Yi-Ching Lee Ph. D

Institute of Cellular and Organismic Biology
Academia Sinica, Taiwan

Below is point-by-point response to the referee's comments.

We thank the reviewers for their thorough and thoughtful evaluation of our original manuscript. We have performed additional experiments and changed the text to address the questions and concerns. New text in the revised manuscript is indicated in blue.

Comments to the Authors:.

Reviewer #1:

The authors claim that GLUT10 mediates adipogenesis by vitamin-C dependent demethylation of DNA. They employed a previously established Glut10G128E mouse strain which carries a human rare variant with compromised GLUT10 function and revealed that the mice show HFD-induced metabolic disorders such as obesity and insulin resistance. Their epidydimal WAT showed reduced weight and increased inflammation and fibrosis, which may cause increased fat storage in the subcutaneous WAT and ectopic lipid accumulation in liver and BAT. Authors also showed that GLUT10 cell-autonomously mediates adipocyte differentiation by presenting that shGlut10 3T3-L1 cells showed reduced adipogenesis as well as reduced uptake of vitamin C. In addition, supplementation of vitamin C induced adipocyte differentiation by mediating mRNA expression which was associated with induced 5hmC levels in the genomic regions of Cebpa and Pparg. Together, the authors conclude that GLUT10 mediates vitamin-C mediated DNA demethylation and gene expression of Cebpa and Pparg to affect adipogenesis. Furthermore, the authors present a clinical link that the SLC2A10 gene which encodes GLUT10 protein is associated with type 2 diabetes mellitus-related phenotypes in non-diabetic Han Taiwanese. The authors' claim is overall convincing, and their findings are very interesting. However, there are several concerns to be addressed as listed.

Response: Thank you very much for the positive feedback.

Points to be addressed

1. Table S5 is missing.

Response: We apologize for omitting Table S5. We have provided it in the revised manuscript.

2. Several figure numbers in the manuscript are incorrectly indicated (i.e. Figure S3A in line 205, Figure S3B in lines 206-207, Figure S3A in line 212). All figure numbers should be precisely indicated.

Response: Thank you for alerting us to these mistakes. We corrected the figure numbers in the revised manuscript.

3. Authors claim in lines 136-139 that "on a HFD, Glut10^{G128}E mice gained more weight than WT mice, despite both genotypes exhibiting comparable food intake, physical activity, energy expenditure, and respiratory exchange ratio (RER) on either a CD or HFD". However, HFD-treated Glut10^{G128E} mice show a clear reduction trend of VO2, VCO2 and heat production compared to other groups in Figure S2. It is suggested to state more precisely that the reduced trend of energy expenditure in HFD-treated Glut10G128E mice would be a cause of their obesity.

Response: Thank you for the comments. We have revised the results section accordingly.

(line,156-158) However, trends toward reductions in physical activity, VO2, VCO2 and heat production were observed in *Glut10*^{G128E} mice on a HFD (Fig. S2B-F).

4. While it is reported that the serum levels of adiponectin are inversely correlated with visceral fat amount, Glut10^{G128E} *mouse shows reduced eWAT with low serum adiponectin level. Authors should discuss the possible mechanisms. For example, if it is considered that the fat accumulation in scWAT, BAT, and ectopic organs affect the phenotype. It is also suggested to discuss the mechanism that GLUT10 and ascorbic acid mainly target eWAT.*

Response: Adiponectin is secreted mainly by adipocytes of WAT, and our data show that its expression is highly induced in 3T3-L1 cells after induction of differentiation (Fig 4K), indicating it is a marker of adipocyte maturation. Additionally, reduced WAT decreases adipokine expression. As such, patients with lipodystrophy exhibit reduced plasma adiponectin and numerous metabolic complications. Interestingly, the reduction of adiponectin levels occurs prior to the onset of obesity and insulin resistance. Several lines of evidence have shown that pro-inflammatory cytokines (i.e., TNF-α and IL-6) reduce adiponectin expression. Together, these results suggest that the inversely correlation of serum adiponectin levels with visceral fat might due to the inflammation. The observations of reduced adipogenesis and increased pro-inflammatory cytokines in *Glut10*^{G128E} eWAT might be responsible for the reduced serum adiponectin levels in *Glut10*^{G128E} mice.

The sizes and weights of sWAT were not significantly difference between WT and *Glut10*^{G128E} mice at 20 weeks of age on CD (Fig 2B and Fig S4A). Although sWAT was increased in *Glut10*^{G128E} mice on HFD, no histological differences with WT were observed (Fig. 2B and S4C), suggesting that the metabolic dysregulation in HFD-fed *Glut10*^{G128E} mice might be mainly attributed to the observed differences in eWAT. Discussion of this topic was added to the revised manuscript.

(line, 401-411) ...a subset of lipodystrophy patients exhibit reduced plasma adiponectin and numerous metabolic complications [57]. Adiponectin is predominantly secreted by adipocytes and plays various protective roles in the body, including anti-inflammatory, antidiabetic, antiatherogenic, and cardioprotective effects [58]. The shGlut10 3T3-L1 cells have reduced adipogenesis and reduced adiponectin levels. Previous reportes showed that serum levels of adiponectin are negatively correlated with visceral fat [59], and several lines of evidence showed that pro-inflammatory cytokines (i.e., TNF-α and IL-6) reduce adiponectin expression [60]. This reduced adiponectin level was observed prior to the onset of obesity and insulin resistance [60]. Thus, the reduced adiponectin in *Glut10*^{G128E} mice might be due to reduced WAT and increased pro-inflammatory cytokines in *Glut10*^{G128E} eWAT.

(line, 421- 424) Although sWAT was increased in *Glut10*^{G128E} mice on HFD, no histological differences with WT were observed, suggesting the metabolic dysregulation in HFD-fed *Glut10*^{G128E} mice might be mainly attributed to eWAT. To this point, significant differences in the functional effects of subcutaneous (sWAT) and visceral fat (eWAT) on metabolic dysfunction have been widely observed [64].

Reviewer #2

In this manuscript, the authors describe the effects of modulating GLUT10 expression or activity and the response in adipose tissue. These studies implicate some effects of GLUT10 in energy metabolism. However, some of the conclusions reached by the authors are reached through implied effects of ascorbic acid in this system, and are poorly supported by their

direct line of questioning. It is recommended that additional data is needed to refer to this phenomenon as 'vitamin C-mediated' as mentioned in the title. A list of specific points that need to be addressed in this manuscript follows.

Major Issues:
1. GLUT10 is present on endomembranes, as the authors note on P13, rather than the plasma membrane. It is unclear how a disruption of this protein results in whole-cell changes in vitamin C transport systems. Cells can transport DHA though various members of GLUT family - many of which are present in 3T3 cells. Thus, the authors should survey all other GLUT members involved in DHA transport to determine if shGlut10 3T3-L1 cells show aberrations in those systems. In other words, is the disruption of GLUT10 shRNA specific?

Respons: Thank you for the comments. GLUT10 is located in the endomembrane system, mitochondria and nuclear envelope, and it functions as a DHA transporter. We have demonstrated that the DHA transported into the endomembrane system and mitochondria can be regenerated into ascorbic acid and enhance cellular uptake of DHA to maintain intracellular ascorbic acid levels. We moved this information to the Introduction in order to make it more clear in the revised manuscript.

We examined the expression of SVCT1 and 2 and all GLUTs in *shLuc* and *shGlut10* 3T3-L1 cells by RT-PCR. Only the expression of GLUT10, GLUT1, GLUT8 and SVCT 2 can be detected in 3T3-L1 cells. The relative expression levels of the expressed transporters were shown below. The targeting sequence of *GLUT10 shRNA,* CCCTGGTTTATTCATCTGCA, specifically targets to GLUT10 and not other murine GLUTs members, according to BLAST results. GLUT10 expression was reduced in *shGlut10* 3T3-L1 cells, whereas we did not observed changes of gene expression in other GLUTs and SVCTs in *shGlut10* 3T3-L1 cells. Discussion about these points was added to the revised manuscript.

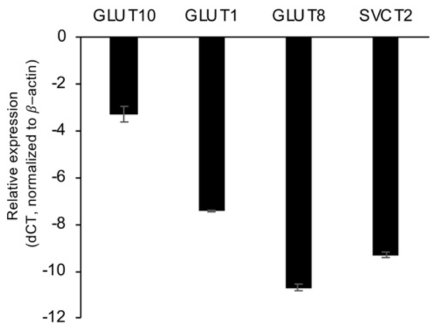

(line 67-72) GLUT10 is localized to the endomembrane system, mitochondria and the nuclear envelope, and it mediates intracellular DHA transport in aortic smooth muscle cells (ASMCs), adipocytes and fibroblasts [14-16, 19]. The DHA transported into subcellular compartments can be regenerated into ascorbic acid, which enhances cellular uptake of DHA as a mechanism to maintain intracellular ascorbic acid levels and redox homeostasis [16-21].

(line,431-437) Among all the known ascorbic acid transporters, only the expression of SVCT2, GLUT10, GLUT1, and GLUT8 can be detected in 3T3-L1 preadipocytes, and the expression levels of GLUT10 and 1 were relatively higher than the SVCT2 and GLUT8. Furthermore, knockdown of GLUT10 expression in *shGlut10* 3T3-L1 cells reduced GLUT10 expression but did not alter the expression of SVCTs and the other GLUTs.

2. Ascorbic acid is transported through SVCT2 in 3T3-L1 cells, yet no attempt was made to determine SVCT2 levels in these cells, especially in the shGlut10 vs. controls. These are

critical experiments (along with those in point #1) to determine if this phenomenon is truly due to changes in GLUT10

Response: We examined the expression of of SVCT1 and 2 in *shLuc* and *shGlut10* 3T3-L1 cells by RT-PCR. Only the expression of SVCT 2 (Ct 28) was detected in 3T3-L1 cells. Its expression was relatively lower than that of GLUT10 (Ct 22) and was unaffected in *shGlut10 3T3-L1* cells. The discussion was added in the discussion section in the revised manuscript.

(line, 431-437) Among all the known ascorbic acid transporters, only the expression of SVCT2, GLUT10, GLUT1, and GLUT8 can be detected in 3T3-L1 preadipocytes, and the expression levels of GLUT10 and 1 were relatively higher than the SVCT2 and GLUT8. Furthermore, knockdown of GLUT10 expression in *shGlut10* 3T3-L1 cells reduced GLUT10 expression but did not alter the expression of SVCTs and the other GLUTs.

3. All of the experiments concerning vitamin C are conducted in 3T3-L1 cells, and no evaluation of the effects of GLUT10 disruption on vitamin C levels, vitamin C transport, or glucose transport were made in the cells isolated from transgenic animals. These are also critical to understanding the effects of GLUT10 disruption, and making comparison from a cell culture model and in vivo disruption. Cells placed in culture are maintained without ascorbic acid and the effects of its addition are unpredictable - in vivo experiments are needed to define physiologically relevant changes.

Response: Thank you for the comments. We compared the intracellular ascorbic acid levels in stromal vascular fraction (SVF) cells freshly isolated from eWAT of WT and *Glut10*^{G128E} mice. The intracellular ascorbic acid levels were decreased in SVF from *Glut10*^{G128E} mice. These results again support the idea that GLUT10 contributes to ascorbic acid homeostasis in mice. These results were included in supplementary Figure S12 and are discussed in the revised manuscript.

(line, 439- 446) Additionally, intracellular ascorbic acid levels were lower in freshly isolated SVF cells from *Glut10*^{G128E} mice compared with SVF cells from WT mice (Fig. S12A)... …….. These results demonstrate that GLUT10 deficiency disturbs ascorbic acid homeostasis in vivo. Mice can endogenously synthesize ascorbic acid in the liver, and serum ascorbic acid levels were significantly increased in *Glut10*^{G128E} mice on HFD (Fig. S12B), suggesting an attempt at compensation for disrupted ascorbic acid homeostasis in *Glut10*^{G128E} mice. The mechanisms contributing to this increased serum ascorbic acid levels in *Glut10*^{G128E} mice are as yet unknown, however the observation supports the idea that GLUT10 deficiency disturbs ascorbic acid homeostasis in vivo.

4. In Fig S4E, serum ascorbate levels in G128E mice appear to be higher than than WT in both feeding conditions, but only significantly higher in the high-fat diet. This would imply that GLUT10 is involved in the biosynthesis of ascorbic acid in the rodent liver or the retention of ascorbic acid in the kidney - yet neither of these phenomenon are discussed in the paper.

Response: Thank you for the comments. Both increased ascorbic acid biosynthesis in the liver and retention of ascorbic acid in the kidney can contribute to increased serum ascorbic acid levels. The expression of GLUT10 was not detected in mouse liver or kidney (Fig. S8). Although we cannot totally exclude the possibility, it is unlikely that GLUT10 contributes to biosynthesis of ascorbic acid in liver or the retention of ascorbic acid in the kidney in *Glut10*^{G128E} mice. However, whether GLUT10 deficiency might alter the expression of genes in liver or kidney and contribute to increased ascorbic acid biosynthesis or retention in

Glut10^{G128E} mice should be further examined. We included the discussion of this topic in the revised manuscript.

(line, 440-446) Mice can endogenously synthesize ascorbic acid in the liver, and serum ascorbic acid levels were significantly increased in *Glut10*^{G128E} mice on HFD (Fig. S12B), suggesting an attempt at compensation for disrupted ascorbic acid homeostasis in *Glut10*^{G128E} mice. The mechanisms contributing to this increased serum ascorbic acid levels in *Glut10*^{G128E} mice are as yet unknown, however the observation supports the idea that GLUT10 deficiency disturbs ascorbic acid homeostasis in vivo.

Minor Issues:
1. Ascorbic acid is not a vitamin for rodents. Since no human cells were used in these experiments, it would be proper to use it chemical name.

Response: Thank you for the comments. We have changed vitamin C to ascorbic acid throughout the revised manuscript; all the changes are indicated in blue.

2. In several parts of the paper, the authors suggest that vitamin C contributes to T2DM, without defining the exact relationship. In some places the wording implies that high vitamin C would promote diabetes, but it the opposite is more likely to be true.

Response: We apologize for the confusion. We have changed the text to make it more clear in the revised manuscript.

(line, 50- 58) Ascorbic acid has been implied to reduce risk of T2DM [6]. Epidemiological studies have shown that plasma ascorbic acid levels are inversely correlated with waist-to-hip ratio [6-9], which reflects the accumulation of visceral fat and is highly associated with the risk of T2DM [10]. Furthermore, animal studies demonstrated that ascorbic acid supplementation reduces inflammation and adiposity in high-fat-diet (HFD)-fed animals [6, 11]. However, clinical trials testing ascorbic acid supplementation for the prevention or treatment of obesity and its co-morbidities have produced inconsistent results [6, 12]. Thus, the possible mechanisms and physiological effects that link ascorbic acid with reduced risk of T2DM require further exploration.

3. The reason that vitamin C is inversely related to body fat composition is due to the increased amount of inflammation/oxidative stress that obesity brings. There is no credible source that suggests vitamin C can reverse obesity alone, but it may mitigate some of its effects.

Response: Thank you for the comments. We have revised the text in the introduction section to better reflect current knowledge and added a sentence in the discussion of the revised manuscript.

(line, 50-55) Epidemiological studies have shown that plasma ascorbic acid levels are inversely correlated with waist-to-hip ratio [6-9], which reflects the accumulation of visceral fat and is highly associated with the risk of T2DM [10]. Furthermore, animal studies demonstrated that ascorbic acid supplementation reduces inflammation and adiposity in high-fat-diet (HFD)-fed animals [6, 11].

(line,486-489) However, whether the disturbance of ascorbic acid homeostasis in *Glut10*^{G128E} mice might affect oxidative stress and inflammation to contribute to the eWAT phenotypes and metabolic consequences should be further explored.

4. The relevant text about GLUT10 being present in endomembranes should be part of the introduction, including text about the other transport mechanisms for vitamin C.

Response: Thank you for the comments**.** We have moved the text from the results section to the introduction section in the revised manuscript.

(line, 60-63) Ascorbic acid and its oxidized form, dehydroascorbic acid (DHA), can be respectively transported into cells and intracellular compartments by active sodium ascorbic acid transporters (SVCTs) and facilitative glucose transporter members (GLUTs), respectively [14].

(line, 67-72) GLUT10 is localized to the endomembrane system, mitochondria and the nuclear envelope, and it mediates intracellular DHA transport in aortic smooth muscle cells (ASMCs), adipocytes and fibroblasts [14-16, 19]. The DHA transported into subcellular compartments can be regenerated into ascorbic acid, which enhances cellular uptake of DHA as a mechanism to maintain intracellular ascorbic acid levels and redox homeostasis [16-21].

5. When the authors state that GLUT10 is associated with diabetes phenotypes, it is unclear if the authors are speaking of normal GLUT10 activity or altered/SNP activity or expression.

Response: We apologize for the confusion. We have have changed the description to "..genetic polymorphisms in *SLC2A10* locus are associated with a T2DM "to make it clear throughout the revised manuscript; all the changes are indicated in blue.

6. On Page 5, line 111-12, the authors imply that GLUT10 controls vitamin C levels in cells - but these results have only been shown in cell culture and show no data from animal or humans to back up this claim.

Response: We added text and references to the Introduction describing that GLUT10 deficiency might affects ascorbic acid levels and may contribute to connective tissue abnormalities in ATS patients and *Glut10*^{G128E} mice.

(line, 65-78) We and others have demonstrated that GLUT10 is a critical factor in the maintenance of intracellular ascorbic acid levels and redox hemostasis in vitro and in vivo [16-20]. …. Ascorbic acid is essential for hydroxylation of prolyl and lysyl residues during collagen synthesis and assembly. The effects of GLUT10 on ascorbic acid-dependent collagen synthesis/assembly and redox balance might explain connective tissues abnormalities observed in mice with functional GLUT10 deficiency and ATS patients [15, 17, 20]

7. Ascorbic acid has effects on 3T3 differentiation that are independent of GLUT10 - it is unclear if the authors have properly attributed changes in gene expression (and 5hmC) with AA supplementation. These should be conducted on cells that have been fully differentiated.

Response: We agree that ascorbic acid can enter 3T3-L1 cells through GLUT10-independent uptake to affect cell differentiation, probably though SVCT2 and GLUT1. Although 3T3-L1 cells express relatively high levels of GLUT10, expression of SVCT2 and GLUT1 can be also detected in 3T3-L1 cells. Nevertheless, our data show that GLUT10 does play a role in maintaining ascorbic acid homeostasis and contribute to DNA demethylation and expression of *Cebpa* and *Pparg* genes in 3T3L1 cells. Upon ascorbic acid supplementation, the intracellular and nuclear ascorbic acid levels remained lower in *shGlut10* 3T3-L1 cells than in *shLuc* 3T3-L1 cells (Fig. 5B). Although ascorbic acid supplementation increased the expression (and demethylation) of *Cebpa* and *Pparg* genes in *shGlut10* 3T3-L1 cells, the gene

expression (and demethylation) remained much lower in *shGlut10* 3T3-L1 cells than in *shLuc* 3T3-L1 cells (Fig. 5E and F; Fig. 6B and C). In fully differentiated 3T3-L1 cells, the gene demethylation and expression of *Pparg* were highly induced (International Journal of Obesity (2017). When we supplemented ascorbic acid in differentiated 3T3-L1 cells (6 days after induction of differentiation) no effect on adipogenesis was observed (Figure below).

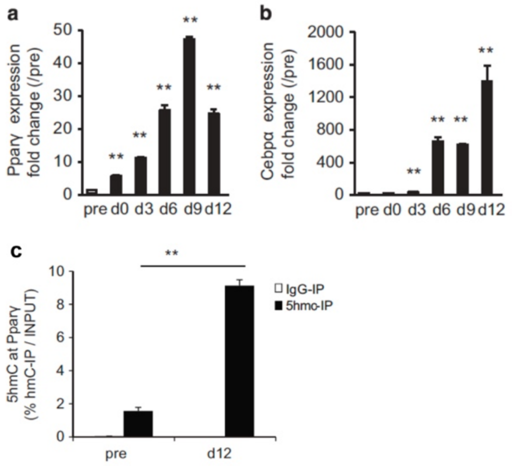

Expression and DNA demethylation of Pparγ gene was highly increased after induction of adipogenic differentiation in 3T3L1 cells. (a and b) The expression of Pparγ and Cebpα was highly upregulated in 3T3 -L1 cells after induction of differentiation, and (c) DNA demethylation at Pparγ gene was also highly increased after induction of adipogenic differentiation. Locus-specific 5-hmC at the Pparγ gene was quantified by hMeDIP (Figures from International Journal of Obesity, 2017; 652 – 659.)

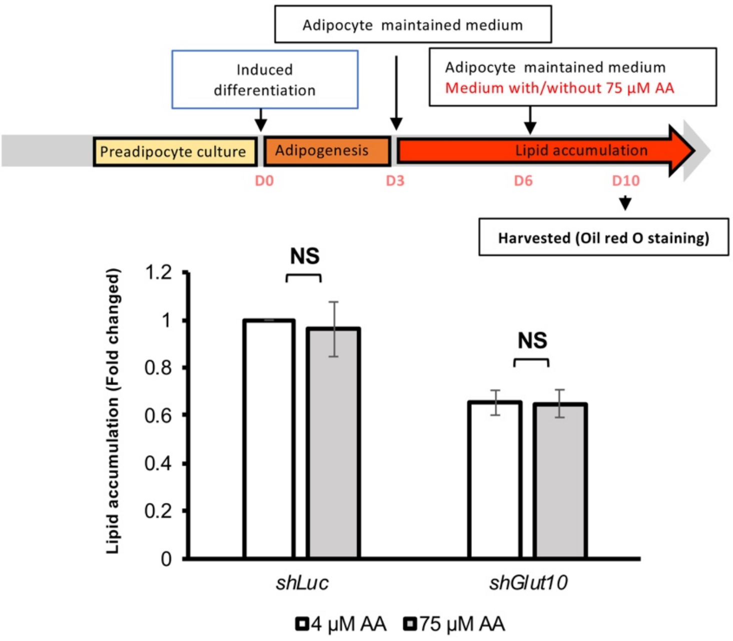

Supplemented ascorbic acid at 6 days after induction of adipogenic differentiation did not affect adipogenesis in 3T3L1 cells (unpublished results).

8. The effects of AA in cell culture can span both pro-oxidant and anti-oxidant effects that are not always apparent in animal systems. These are typically thoughts of as cell culture artifacts due to the high oxygen and iron content in cell culture systems. Besides performing these experiments in isolated issues from animals, it is suggested that the authors attempt to determine if changes in vitamin C levels are reducing or increasing ROS burden. Also, the increased ROS production by GLUT10 changes (or other glucose regulation) could be responsible for these apparent effects on vitamin C, and should be explored further.

Response: Thank you for the suggestion. The intracellular and mitochondrial ROS levels were increased in *shGlut10* 3T3-L1 cells (Fig S14 A and B). Pre-incubating *shGlut10* 3T3-L1 cells with ascorbic acid or the antioxidants, NAC (*N*-acetyl-cysteine) and MitoQ (mitochondria-targeted coenzyme Q10), reduced intracellular and mitochondrial ROS levels (Fig S14 A and B). Importantly, however, only ascorbic acid, but not the other antioxidants, could enhance adipogenesis in *shGlut10* 3T3-L1 cells (Fig S14 C and D). Furthermore, the intracellular and mitochondrial ROS levels were relatively low in *shLuc* 3T3-L1 cells, and while ascorbic acid supplementation did not further reduce the intracellular or mitochondrial ROS levels in these cells, it did promote adipogenesis (Fig S14 C and D). These results suggest that ascorbic acid promotion of adipogenesis in 3T3L1 cells might not depend on its free radical scavenger function. We agree that AA effects can be different in cell culture system and in animal systems. The disturbances in ascorbic acid hemostasis might affect oxidative stress and inflammation and contributes to the eWAT phenotypes of *Glut10*^{G128E} mice. We discussed the concern about differences between cell culture and animal systems with regard to ROS in the revised manuscript.

(line, 473-489) It is well established that ascorbic acid is an antioxidant/free radical scavenger. We further explore the possibility of antioxidant roles of ascorbic acid in adipogenesis. Consistent with the decreased intracellular ascorbic acid level, intracellular and mitochondrial reactive oxygen species (ROS) levels were increased in *shGlut10* 3T3-L1 cells (Fig. S15A and B). Pre-incubating *shGlut10* 3T3-L1 cells with ascorbic acid or the antioxidants NAC (*N*-acetyl-cysteine) and MitoQ (mitochondria-targeted coenzyme Q10) reduced intracellular or mitochondrial ROS levels (Fig. S15A and B). Importantly, however, only ascorbic acid, but not the other antioxidants, could enhance adipogenesis in *shGlut10* 3T3-L1 cells (Fig. S15C and D). Furthermore, the intracellular and mitochondrial ROS levels were relatively low in *shLuc* 3T3-L1 cells, and whereas ascorbic acid supplementation did not further reduce the intracellular or mitochondrial ROS levels in these cells, it did promote adipogenesis (Fig. S15C and D). These results suggest that ascorbic acid promotes adipogenesis in 3T3L1 cells might not through its free radical scavenger function. However, whether the disturbed ascorbic acid homeostasis in *Glut10*^{G128E} mice might affect oxidative stress and inflammation and contributes to the eWAT phenotypes and metabolic consequences should be further explored.
