# Supplementary material for: Glucose transporter 10 modulates adipogenesis via an ascorbic acid-mediated pathway to protect mice against diet-induced metabolic dysregulation
Source: PLoS Genet. 2020 May 26;16(5):e1008823. doi: 10.1371/journal.pgen.1008823 (PMC7274451; doi:10.1371/journal.pgen.1008823)
Supplement: Attachment — Submitted filename: 20200428 PLOS GLUT10 cover letters.pdf [file pgen.1008823.s022.pdf]

April 28, 2020

PLOS Genetics (PGENETICS-D-20-00046R1)
Title: Glucose transporter 10 modulates adipogenesis via an ascorbic acid-mediated pathway to protect mice against diet-induced metabolic dysregulation

Gregory S. Barsh
Gregory Copenhaver
Editor-in-Chief
PLOS Genetics

Dear Drs. Barsh and Copenhaver,

We appreciate the careful review by reviewers again. We have addressed the concerns from reviewer #2 in the revised manuscript. Please find the point-by-point response to the referee's comments and a description of the changes made in the revised manuscript below.

We are happy to submit the revised manuscript for your consideration.

With kind regards,
Yi-Ching Lee Ph. D

Institute of Cellular and Organismic Biology
Academia Sinica, Taiwan

The point-by-point response to the referee's comments below.

1. In their response, the authors claim that other vitamin C transport proteins (GLUTs and SVCT2) are not affected by the knockdown of GLUT10. This data, however, is not provided. Given the wealth of other data that the authors provided, this omission is notable. Can this be included?

Response: We have included the expression data of GLUTs and SVCTs in *shLuc* and *shGlut10* 3T3-L1 cells in supplementary data.

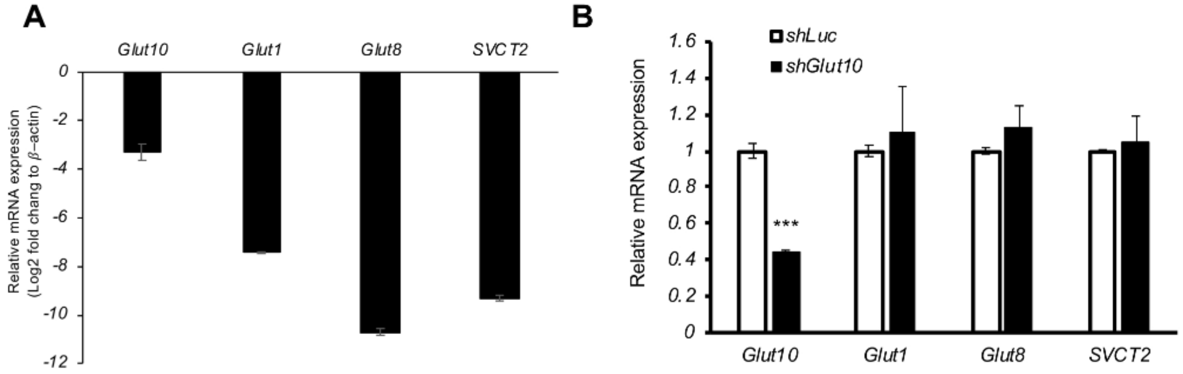

S9 Fig. The expression of SVCTs and GLUTs in *shLuc* and *shGlut10* 3T3-L1 cells. The mRNA expression levels of GLUTs and SVCTs in *shLuc* and *shGlut10* 3T3-L1 cells were analyzed by RT-PCR. (A) Only the expression of GLUT10, GLUT1, GLUT8 and SVCT 2 can be detected in 3T3-L1 cells. The relative expression of the expressed transporters was shown in dCT values normalized to β-actin expression. (B) Knockdown of GLUT10 expression did not alter the expression of other transporters. The expression levels of the transporters in *shGlut10* 3T3-L1 cells were compared to those in *shLuc* 3T3-L1 cells. n = 3 independent experiments. Data are shown as the mean ± SEM. *P < 0.05, **P < 0.01, ***P < 0.001.

2. The data with AA uptake by the cells in Figure 5B appears to indicate that the GLUT10 knockdown cells are unable to transport AA at the same rate they did in the WT cells. However, given that this experiment was conducted over a time and cell culture condition where ascorbate could oxidize to DHA, this could be attributable to changes in DHA transport alone. Either the Figure and the corresponding text needs to be changed to altered to reflect this, or the experiment needs to be repeated under conditions where AA transport alone is measured.

Response: We have revised the corresponding text of Figure 5B to clarify the issue in revised manuscript.

(line 319-330) Ascorbic acid could be transported into cells in its reduced form or oxidized form by SVCTs or GLUTs respectively. GLUT10 was expressed at relatively higher levels in 3T3-L1 preadipocytes compared to other expressed transporters (Fig S9A). Knockdown of GLUT10 expression did not alter the expression of other transporters (Fig S9B). As expected, DHA uptake and intracellular ascorbic acid levels were reduced in *shGlut10* 3T3-L1 cells cultured in 75 μM DHA (Fig 5A). Cultured the cells in a physiological concentration of ascorbic acid (75 μM) where ascorbic acid could be oxidized to DHA, the intracellular and

nuclear ascorbic acid levels were also reduced in *shGlut10* 3T3-L1 cells (Fig 5B). These results demonstrate that GLUT10 maintains intracellular ascorbic acid levels in 3T3-L1 preadipocytes under physiological conditions.
